# Import mechanism of peroxisomal proteins with an N-terminal signal sequence

**DOI:** 10.1038/s41556-025-01662-5

**Published:** 2025-05-09

**Authors:** Michael L. Skowyra, Tom A. Rapoport

**Affiliations:** 1 Howard Hughes Medical Institute and Department of Cell Biology, Harvard Medical School, Boston, MA USA; 2 Present Address: Department of Biochemistry, University of Texas Southwestern Medical Center, Dallas, TX USA

**Keywords:** Protein translocation, Peroxisomes, Mechanisms of disease, Chaperones, Synthetic biology

## Abstract

Most proteins imported into peroxisomes use a carboxy-terminal PTS1 signal, which is recognized by soluble receptors that transport the cargo through a nuclear pore-like conduit in the peroxisomal membrane formed by the tyrosine and glycine-rich YG domain of PEX13. The receptors then return to the cytosol through a separate retrotranslocon. Some peroxisomal proteins instead use an amino-terminal PTS2 signal that is recognized by an adaptor called PEX7, but how they are imported is poorly understood. Here we show that PTS2 cargo is moved through the YG phase by PEX7 bound to a receptor. After cargo release inside peroxisomes, PEX7 returns to the cytosol by moving back on its own through the YG phase. The chaperone PEX39 then extracts PEX7 from the phase on the cytosolic side and helps to reload PEX7 with a new receptor and cargo to start another import cycle. Our results provide a comprehensive model of PTS2 protein import.

## Abstract (web summary)

Skowyra and Rapoport find that peroxisomal proteins with N-terminal PTS2 signals are imported through a nuclear pore-like conduit by PEX7 and a receptor. PEX7 then returns through the same conduit and is extracted into the cytosol by the chaperone PEX39.

## Main

Peroxisomes are conserved organelles essential for human health. They consist of a single membrane enclosing a luminal matrix^1^ and play crucial metabolic roles that include fatty-acid β-oxidation and ether-lipid biosynthesis^2^. Peroxisomal matrix proteins are made in the cytosol and then imported, remarkably, in a folded state and even as oligomers or preformed complexes^3^, in contrast to the endoplasmic reticulum and mitochondria that can only import unfolded polypeptides^4,5^.

Most peroxisomal matrix proteins contain a type 1 peroxisomal targeting signal (PTS1) that is located at their carboxy (C) terminus and comprises the amino-acid sequence SKL (or variants thereof)^6^. Some matrix proteins, however, use the more complex PTS2 signal located at their amino (N) terminus^7^. PTS2 proteins include the enzyme thiolase required for peroxisomal β-oxidation in nearly all eukaryotes^8^ as well as enzymes that detoxify branched-chain fatty acids and produce ether-lipid precursors essential for myelination^2^. Defective PTS2 protein import in humans causes Refsum disease^9^ and the life-threatening disorder rhizomelic chondrodysplasia punctata^10^.

The import mechanism of PTS1 proteins is reasonably well understood^11^. Their PTS1 signal is recognized in the cytosol by the tetratricopeptide-repeat (TPR) domain of PEX5 (ref. ^12^) and related soluble receptors^13,14^. PEX5 then transports the cargo completely across the peroxisomal membrane^15,16^ through a conduit formed by the tyrosine and glycine-rich YG repeats of the PEX13 membrane protein^17,18^. The conduit resembles the selective phase formed by the phenylalanine and glycine-rich nucleoporin FG repeats in nuclear pores^19^ and can be reconstituted in vitro as a hydrogel into which PEX5 can drag folded cargo^17^. Passage through the phase requires conserved motifs in PEX5 comprising the amino-acid sequence WXXXF/Y (where ‘X’ denotes any residue)^17^. PEX5 returns to the cytosol through a separate channel formed by a membrane-embedded ubiquitin ligase complex^20^. The receptor’s flexible N terminus inserts into this retrotranslocon and gets mono-ubiquitinated on a conserved cysteine; ubiquitinated PEX5 is then pulled out of the organelle by a cytosolic AAA ATPase^21^. Pulling unfolds the TPR domain and thereby strips off the cargo^15^. In the cytosol, PEX5 is deubiquitinated^22,23^ and presumably also refolds before starting another import cycle.

PTS2 protein import is by contrast poorly understood. The PTS2 signal is recognized by an adaptor called PEX7 that anchors the cargo to a compatible receptor^24^. In humans and other animals, the receptor is a specific splice variant of PEX5 called the long isoform (PEX5L); a shorter isoform (PEX5S) lacks the PEX7-binding region^25,26^. In yeast, PEX7 associates instead with specialized receptors such as PEX18 and PEX21 that resemble PEX5L but lack a TPR domain^27^. PEX7 shuttles the bound cargo and receptor completely across the peroxisomal membrane^28–35^. The mechanism, however, remains to be established because PEX7 lacks the WXXXF/Y motifs required for traversing the YG phase; whether the receptors facilitate translocation or simply stabilize the interaction with cargo is not clear. How PEX7 returns from the matrix to the cytosol is also unknown. PEX7 consists of WD40 repeats that form a seven-bladed β-propeller^24^ and has neither a flexible N terminus for insertion into the retrotranslocon nor a conserved cysteine for mono-ubiquitination. PEX7 therefore cannot be recycled like PEX5 and other receptors.

## Results

### PTS2 protein import through a YG phase

**Fig. 1:**
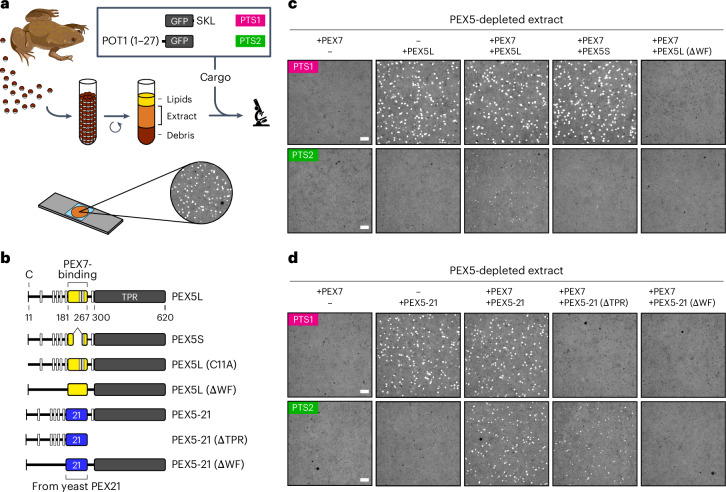
PTS2 protein import reconstituted in *Xenopus* egg extract. **a**, *Xenopus* egg extract was incubated with fluorescent cargo, mounted on a slide and imaged using a spinning-disk confocal microscope. Cargo consisted of GFP fused to the PTS1 signal SKL or the PTS2 signal from the yeast peroxisomal thiolase POT1 (residues 1–27). Repeated rounds of cargo import into peroxisomes produce bright puncta. Created with BioRender.com. **b**, Schematic of the PEX5 variants used. Numbers refer to residues in *Xenopus* PEX5L. Black bar, position of the conserved cysteine (C); white bars, locations of WXXXF/Y motifs; yellow box, PEX7-binding region. PEX5S lacks most of the PEX7-binding region, whereas in PEX5-21 the region was replaced by the homologous region from the yeast PTS2 receptor PEX21. **c**, Extract was depleted of endogenous PEX5 using beads conjugated to the PEX5-binding domain from PEX14 and then supplemented with or without purified recombinant yeast PEX7 and the indicated purified recombinant PEX5 variants (described in **b**). Import was assessed using the fluorescent PTS1 and PTS2 cargos described in **a**. **d**, As in **c** except using the PEX5 variants shown. Scale bars, 5 µm.

**Extended Data Fig. 1:**
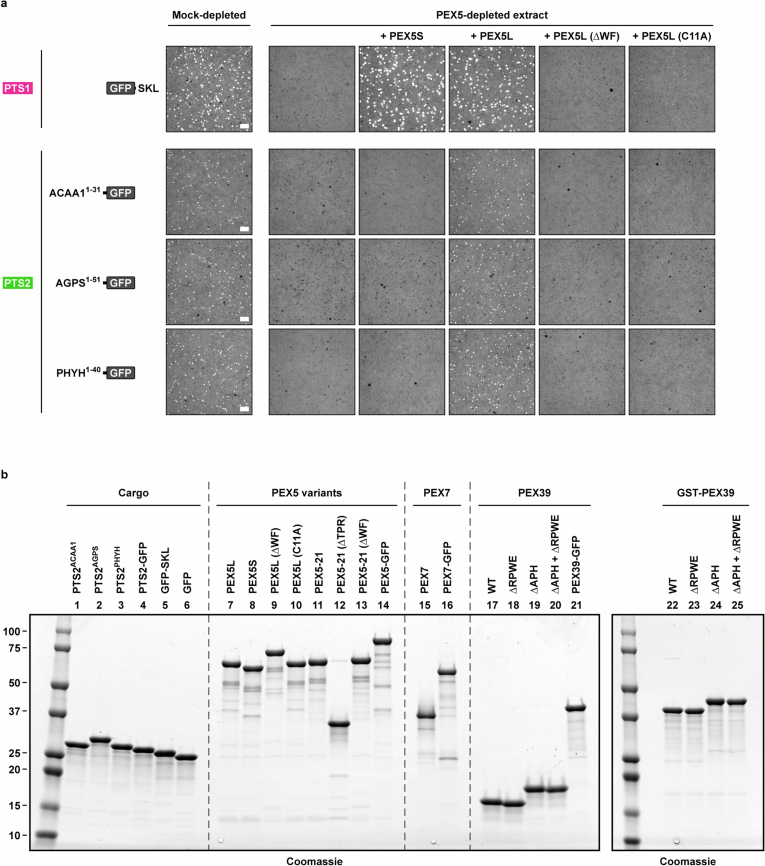
Peroxisomal protein import reactions in *Xenopus* egg extract and purity of recombinant proteins used in this study. **a**, *Xenopus* egg extract was depleted or not (mock) of endogenous PEX5 using beads conjugated to the PEX5-binding domain from PEX14, then supplemented with buffer or the indicated purified recombinant PEX5 variants (described in Fig. 1b). Import was assessed by imaging the indicated fluorescent cargo on a spinning-disk confocal microscope. The cargo, shown on the left, consisted of GFP fused to the SKL peroxisome targeting signal (PTS1) or to the signal from the indicated *Xenopus* PTS2 peroxisomal matrix enzymes (PTS2). Repeated rounds of cargo import into peroxisomes produce bright puncta. Scale bars equal 5 µm. **b**, The indicated recombinant proteins were produced and purified as described in the Methods, then resolved by SDS–PAGE and stained by Coomassie Blue. Molecular weights (in kD) are marked on the left; images of the unprocessed gels are provided. Source data

To understand how PTS2 proteins are brought into peroxisomes, we reconstituted the process using a cytoplasmic extract from the eggs of the frog *Xenopus laevis*. As shown previously^15,36,37^, this cell-free system recapitulates the import of PTS1 cargo such as green fluorescent protein (GFP) fused to an SKL sequence: over time the cargo accumulates inside peroxisomes that then become visible as bright puncta under a microscope (Fig. 1a). To adapt this system for PTS2 cargo, we fused GFP to the import signal from different *Xenopus* PTS2 proteins (Extended Data Fig. 1a; purity of all recombinant proteins in Extended Data Fig. 1b). Each PTS2 cargo formed puncta in the extract (Extended Data Fig. 1a), demonstrating a functional PTS2 import pathway. The puncta were dimmer than those formed by PTS1 cargo, suggesting that PTS2 import is less efficient. As expected, PTS2 import was abolished following depletion of PEX5 from the extract (Extended Data Fig. 1a) and could be restored with purified recombinant PEX5L, the long isoform that can bind to PEX7 (schematic of the PEX5 variants used in Fig. 1b) but not with the short isoform PEX5S that cannot bind (Extended Data Fig. 1a). Either isoform could restore PTS1 import (Extended Data Fig. 1a). The PTS2 puncta result from multiple import cycles because they were not visible with a PEX5 mutant lacking the conserved cysteine (C11A), which cannot be mono-ubiquitinated and recycled (Extended Data Fig. 1a). Notably, both PTS2 and PTS1 import was abolished by a PEX5 mutant lacking the WXXXF/Y motifs (∆WF) that enable translocation through the YG phase (Extended Data Fig. 1a).

**Extended Data Fig. 2:**
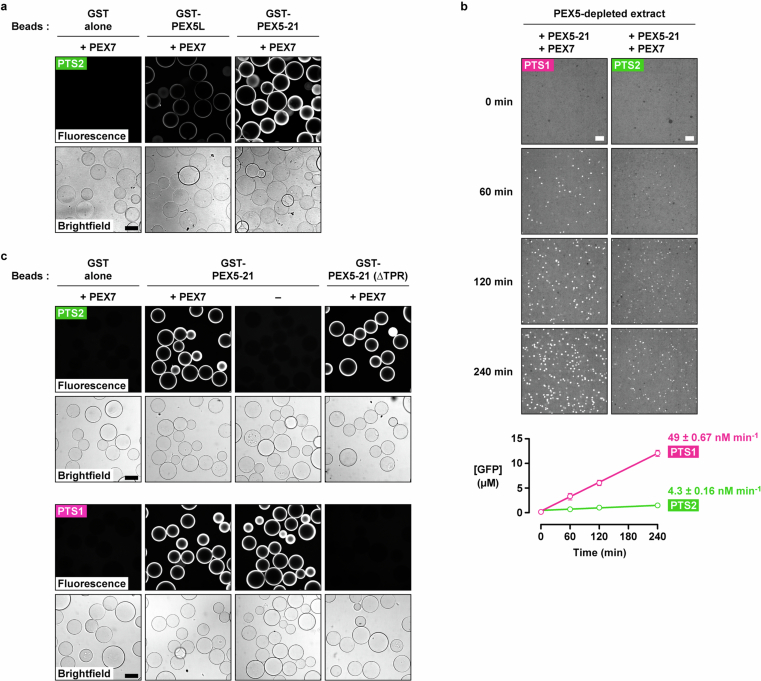
Interaction of PEX5 variants with cargo and kinetics of peroxisomal protein import in *Xenopus* egg extract. **a**, Glutathione-conjugated beads were bound to 1 µM GST alone or to the purified recombinant GST-tagged PEX5 variants shown (described in Fig. 1b), and incubated in the presence of 1 µM purified recombinant yeast PEX7 and 1 µM GFP fused at the N terminus to the import signal (residues 1–27) from yeast POT1 (PTS2). Recruitment of the fluorescent cargo to the beads was visualized after 1 h on a spinning-disk confocal microscope as a bright ring around the bead perimeter. Scale bar equals 100 µm. **b**, *Xenopus* egg extract was depleted of endogenous PEX5 using beads conjugated to the PEX5-binding domain from PEX14, then supplemented with the chimeric receptor PEX5-21 (described in Fig. 1b) and purified recombinant yeast PEX7. Import was assessed using fluorescent PTS1 and PTS2 cargo: the PTS2 cargo is described in panel **a**, whereas the PTS1 cargo consisted of GFP fused at the C terminus to the SKL peroxisome targeting signal. Reactions were imaged at the indicated times on a spinning-disk confocal microscope. The fluorescence intensity of the resulting puncta (corresponding to imported cargo) was then measured, converted into GFP concentrations as described in the Methods, and plotted as a function of time. Error bars span the range of at least 30 imaged fields; lines represent linear fits to the data generated by least-squares regression. The corresponding import rates (±the standard error) are specified beside the graph. Scale bars equal 5 µm. **c**, As in panel **a**, using GST alone or the indicated GST-tagged PEX5 variants (described in Fig. 1b), in the presence or absence of purified recombinant yeast PEX7 and the fluorescent cargo described in panels **a** and **b**. Source numerical data are provided. Source data

We next assessed the importance of PEX7. Because frog PEX7 was unstable when produced recombinantly, we instead used yeast PEX7 and cargo consisting of GFP fused to the PTS2 signal from the yeast peroxisomal thiolase POT1 (Fig. 1a). Yeast PEX7 together with PEX5L (but not PEX5S) could restore import of the PTS2 cargo in PEX5-depleted extract (Fig. 1c), albeit inefficiently, probably reflecting the relatively weak interaction between PEX5L and yeast PEX7 (Extended Data Fig. 2a). To improve the affinity of the receptor for yeast PEX7, we replaced the PEX7-binding region in PEX5L with the homologous region from the yeast PTS2 receptor PEX21 (Fig. 1b). The resulting chimaeric receptor, PEX5-21, bound to yeast PEX7 more strongly (Extended Data Fig. 2a) and boosted import of the PTS2 cargo (Fig. 1d). Both the receptor and yeast PEX7 were necessary for import (Fig. 1d). The import rate of the PTS2 cargo was notably slower than that of PTS1 cargo (Extended Data Fig. 2b). A receptor mutant lacking the TPR domain could still bind to PEX7 and PTS2 cargo as expected (Extended Data Fig. 2c) as well as import the cargo into peroxisomes in egg extract (Fig. 1d). Importantly, PTS2 import by either PEX5L or PEX5-21 again depended on the WXXXF/Y motifs of the receptor (Fig. 1c,d), providing support for the hypothesis that PTS2 proteins are moved into peroxisomes through the YG conduit.

**Fig. 2:**
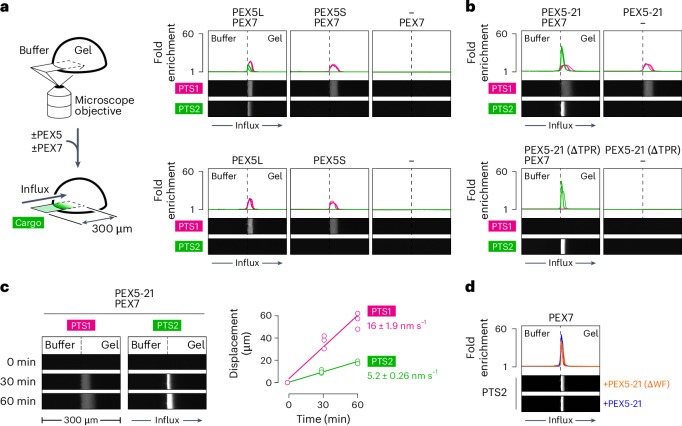
PTS2 cargo enters peroxisomes through a YG phase. **a**, YG hydrogels were incubated with the fluorescent cargo shown (described in Fig. 1a) with or without purified recombinant yeast PEX7 and the indicated recombinant PEX5 variants (described in Fig. 1b). Cargo accumulation inside the gel was visualized on a point-scanning confocal microscope as a bright zone expanding inwards from the gel edge (left). Representative images after a 30-min incubation (right). The fold enrichment of cargo relative to buffer, across the imaged field, for each of three independent experiments is plotted above the images. The width of each image represents 300 µm. **b**, As in **a** but in the presence of the indicated PEX5 variants with or without PEX7. **c**, As in **a** using PEX5-21 and PEX7. Representative images after the indicated times (left). Width of the permeation zone (displacement) in each of three independent experiments as a function of time (right). Linear fits to the data generated by least-squares regression yield the corresponding permeation rates (mean ± s.e.m.). **d**, As in **a** using PTS2 cargo in the presence of PEX7 and the indicated PEX5 variants. Source numerical data are provided. Source data

**Extended Data Fig. 3:**
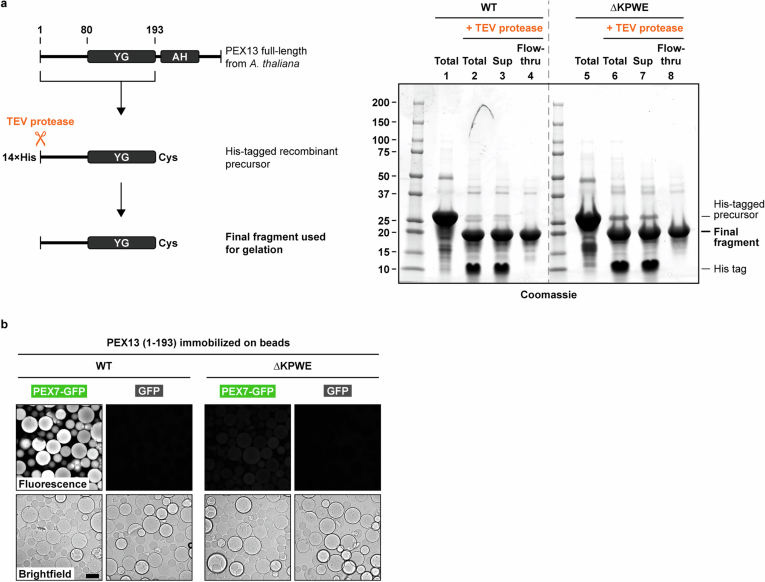
Purification of recombinant YG-domain polypeptides used to prepare YG hydrogels. **a**, Left, scheme depicting the domain organization of PEX13 from *Arabidopsis thaliana*. Note that *A. thaliana* PEX13 lacks the transmembrane segment and SH3 domain found in metazoan PEX13. Relevant residue numbers are specified on top, and the region that was produced recombinantly to prepare YG hydrogels is underlined beneath. The recombinant fragment was fused at the N terminus to a tetradecahistidine (14×His) tag and a TEV protease-cleavage site, and at the C terminus to a cysteine (Cys) residue. Right, purification of the wild-type fragment (WT) and the version lacking the KPWE motif (∆KPWE). Lanes 1 and 5 show each His-tagged precursor purified by nickel-affinity chromatography in the presence of urea (as described in the Methods), resolved by SDS–PAGE and stained by Coomassie blue. Lanes 2 and 6 show each fragment after cleavage with TEV protease to release the His tag. Lanes 3 and 7 show the soluble fraction (sup) of each TEV-cleavage reaction, demonstrating that each fragment remains soluble in urea in the absence of the tag. The cleaved tag was removed from the reactions by a subsequent nickel-affinity step, and the resulting flow-through containing each final purified fragment is shown in lanes 4 and 8. Molecular weights (in kD) are marked on the side; an image of the unprocessed gel is available in source data. **b**, Thiol-reactive beads were conjugated to the purified wild-type (WT) YG-domain polypeptide or the version lacking the KPWE motif (∆KPWE) described in panel **a**. The beads were then incubated with PEX7 fused at the C terminus to GFP (PEX7–GFP) or with GFP alone, and binding of each fluorescent protein to the immobilized fragments was visualized on a spinning-disk confocal microscope. Scale bar equals 100 µm. Source data

To test whether PTS2 proteins are shuttled through the YG phase, we reconstituted the phase as a hydrogel using a recombinant fragment of PEX13 comprising the YG domain and upstream unstructured region (Extended Data Fig. 3a). As shown previously^17^, entry of fluorescently labelled proteins into the phase can be visualized under a microscope as a bright zone expanding inwards from the gel edge (Fig. 2a). Consistent with the egg extract experiments, PTS2 cargo accumulated in the YG hydrogel in the presence of PEX7 and the long receptor isoform PEX5L, whereas no entry was observed with either component alone or with the short isoform (Fig. 2a). By contrast, either isoform could drag PTS1 cargo into the gel independently of PEX7 (Fig. 2a), as expected. PTS2 entry was enhanced by the chimaeric receptor PEX5-21, consistent with its increased affinity for PEX7, whereas the efficiency of PTS1 entry remained unchanged (Fig. 2b). PTS2 entry also did not depend on the receptor’s TPR domain, whereas PTS1 entry did (Fig. 2b). Thus, PTS2 proteins are probably translocated into peroxisomes through the YG phase, and PEX5 and PEX7 are both critical for this process. Interestingly, PTS2 cargo was dragged through the YG hydrogel more slowly than PTS1 cargo (Fig. 2c), in agreement with the slower rate of PTS2 import measured in egg extract. In addition, the entry of PTS2 cargo into the gel was not greatly affected by ablation of the receptor’s WXXXF/Y motifs (Fig. 2d), which are otherwise essential for moving PTS1 cargo through the YG phase^17^. The PTS2–cargo complex therefore probably interacts with the YG phase not just through the receptor.

### PEX7 partitions into the YG phase

**Fig. 3:**
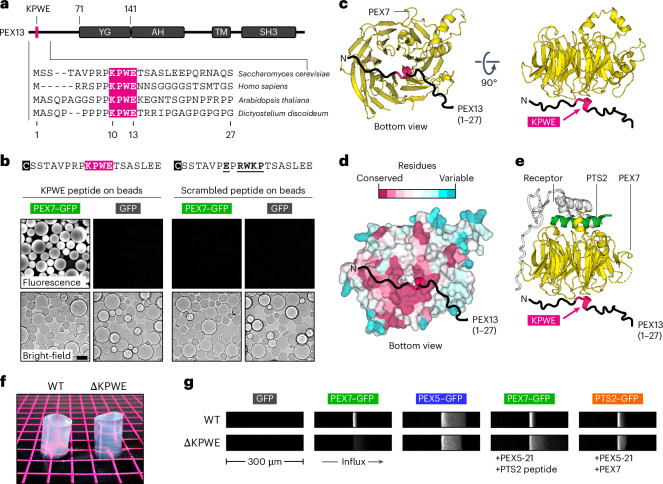
PEX7 enters the YG phase. **a**, Domain organization of yeast PEX13 showing the YG domain, amphipathic helix (AH), transmembrane segment (TM) and SH3 domain. The horizontal line represents unstructured regions. The conserved KPWE motif is coloured pink in the amino-acid sequence alignment (bottom; UniProt ID: P80667, Q92968, Q9SRR0 and Q54CL3). The numbered residues correspond to yeast PEX13. **b**, Thiol-reactive beads were conjugated to a cysteine-containing synthetic peptide comprising the KPWE motif of yeast PEX13 or a peptide in which the relevant residues (underlined) were scrambled. The N-terminal cysteine used for conjugation is shaded. Beads were incubated with yeast PEX7 fused to GFP (PEX7–GFP) or GFP alone; recruitment to the beads was visualized after 1 h on a spinning-disk confocal microscope. Scale bar, 100 µm. **c**, AlphaFold model of the complex between yeast PEX7 and the indicated N-terminal segment of yeast PEX13 containing the KPWE motif. **d**, As in **c** with PEX7 represented as a surface and coloured according to the indicated amino-acid conservation scale. **e**, As in **c** but overlaid on the crystal structure (PDB ID: 3W15) of yeast PEX7, the PTS2 signal and the yeast PTS2 receptor PEX21. **f**, Hydrogels formed from a recombinant fragment of PEX13 comprising the wild-type N-terminal unstructured region and YG domain (WT) or an analogous fragment lacking the KPWE motif (∆KPWE). **g**, Hydrogels as in **f** were incubated with the indicated fluorescent proteins and components. The accumulation of each fluorescent protein inside the gel was visualized on a point-scanning confocal microscope after 1 h. The width of each image corresponds to 300 µm.

A possible additional interaction of the PTS2–cargo complex with the YG phase is suggested by the reported binding of PEX7 to the unstructured region of PEX13 preceding the YG domain^38^. We identified within this region a conserved motif characterized by the amino-acid sequence KPWE (Fig. 3a). Beads conjugated to a synthetic peptide containing this motif could recruit yeast PEX7 fused at the C terminus to GFP (PEX7–GFP), whereas a peptide with the motif scrambled could not (Fig. 3b). PEX7–GFP was also recruited to beads conjugated to a polypeptide encompassing the entire unstructured region and YG domain but only when the KPWE motif was intact (Extended Data Fig. 3b). The KPWE motif thus directly binds to PEX7 and is the only PEX7-binding site within the region of PEX13 that forms the YG phase.

**Extended Data Fig. 4:**
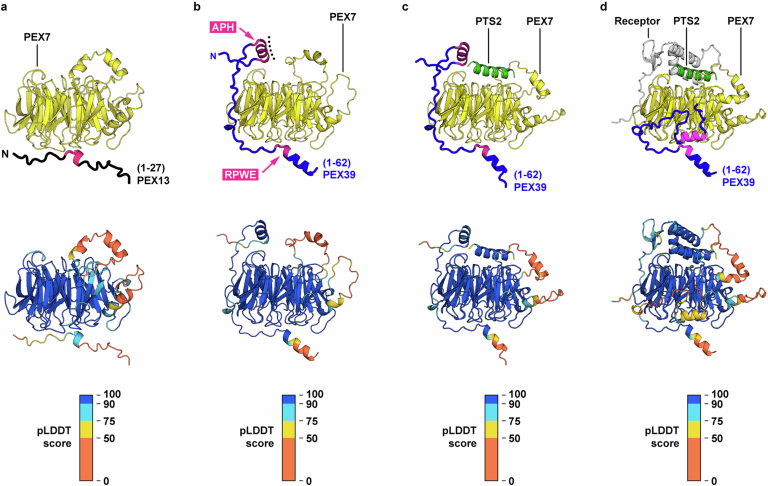
Confidence scores of predicted PEX7 complexes. **a**, Top, AlphaFold model from Fig. 3c of the complex between yeast PEX7 and the N-terminal segment of yeast PEX13 (residues 1–27) containing the KPWE motif. Below, the model coloured according to the predicted local distance difference test (pLDDT) score computed by AlphaFold for each residue, with a score of 100 reflecting the highest confidence. **b**, As in panel **a**, showing the AlphaFold model from Fig. 5b of the complex between yeast PEX7 and the region of yeast PEX39 that contains the APH and RPWE motif. **c**, As in panel **a**, showing the AlphaFold model from Fig. 7a of the complex between yeast PEX7 and the region of yeast PEX39 that contains the APH and RPWE motif, in the presence of a PTS2 peptide. **d**, As in panel **a**, showing the AlphaFold model from Fig. 7a of the complex between yeast PEX7 and the region of yeast PEX39 that contains the APH and RPWE motif, in the presence of a PTS2 peptide and the yeast receptor PEX21.

**Extended Data Fig. 5:**
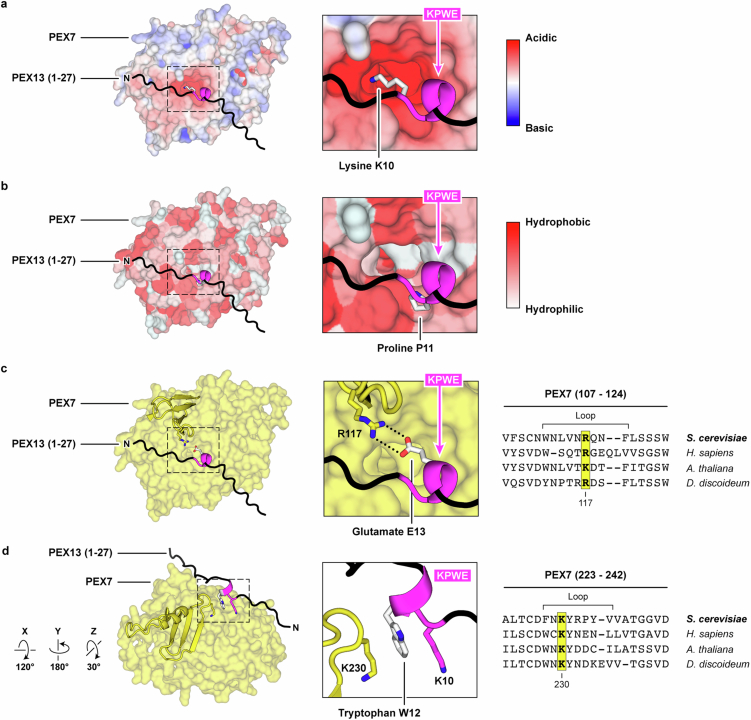
Structural modelling of the interaction between PEX7 and the KPWE motif of PEX13. **a**, Interaction between full-length yeast PEX7 and residues 1–27 (including the KPWE motif) of yeast PEX13, predicted by AlphaFold. PEX7 is shown as a space-filling model in the same orientation as in Fig. 3c, coloured by surface electrostatic potential according to the scale on the right. The PEX13 fragment is depicted as a ribbon, with the side chain of lysine 10 (K10) of the KPWE motif shown. The region outlined by a dashed black line is magnified on the right. **b**, As above, with PEX7 coloured by surface hydrophobicity according to the scale on the right, and showing the side chain of proline 11 (P11) of the KPWE motif. **c**, As above, with PEX7 coloured yellow and showing the side chain of glutamate 13 (E13) of the KPWE motif. E13 is predicted to contact arginine 117 (R117) within the loop of one of the WD40 blades of the PEX7 β-propeller (overlaid on top as a cartoon representation). A sequence alignment of this loop from the indicated organisms is shown on the right, illustrating the conservation of the arginine residue. **d**, As in panel **c**, with the model rotated as indicated and showing the side chain of tryptophan 12 (W12) of the KPWE motif. W12 is predicted to contact the aliphatic stem of lysine 230 (K230) within the loop of one of the WD40 blades of the PEX7 β-propeller (overlaid on top as a cartoon representation). A sequence alignment of this loop from the indicated organisms is shown on the right, illustrating the conservation of the lysine residue.

**Extended Data Fig. 6:**
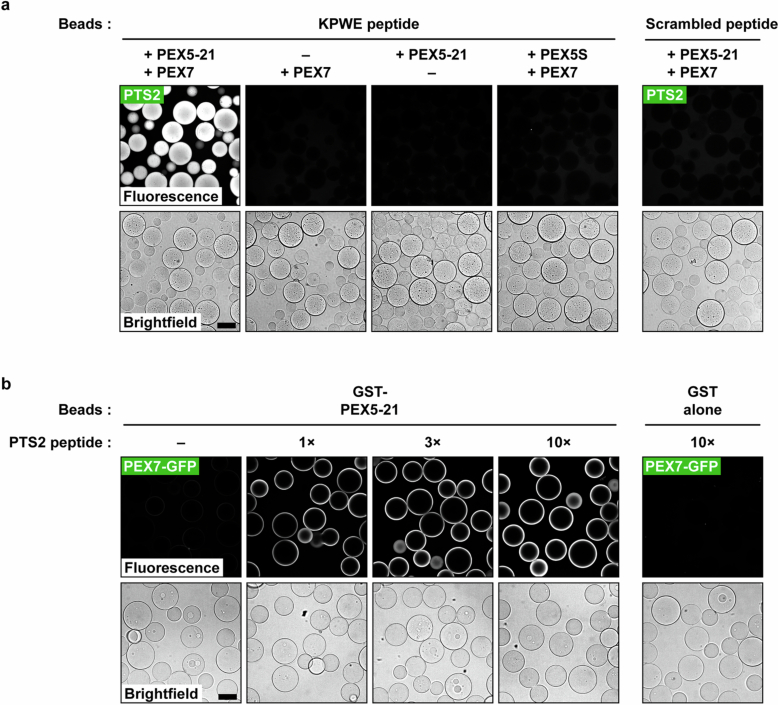
Interaction between PEX7 and the KPWE motif of PEX13. **a**, Thiol-reactive beads were conjugated to a cysteine-containing synthetic peptide comprising the region of yeast PEX13 that contains the KPWE motif, or a peptide in which the motif was scrambled (both described in Fig. 3b). The beads were then incubated with or without 1 µM yeast PEX7, in the presence of 1 µM cargo consisting of GFP fused at the N terminus to the import signal (residues 1–27) from yeast POT1 (PTS2). Where indicated, reactions included 1 µM of either the chimeric receptor PEX5-21 (described in Fig. 1b) that can bind to PEX7, or the short receptor isoform PEX5S that cannot bind. Recruitment of the cargo to the beads after 1 h was visualized on a spinning-disk confocal microscope. Scale bar equals 100 µm. **b**, Glutathione-conjugated beads were bound to the purified recombinant GST-tagged chimeric receptor PEX5-21 (described in Fig. 1b) or to GST alone. The beads were then incubated with 1 µM PEX7–GFP in the absence or presence of a synthetic peptide comprising the PTS2 signal (residues 2–15) from yeast POT1; the fold excess of the peptide relative to PEX7–GFP is indicated above. Recruitment of PEX7–GFP to the beads was visualized on a spinning-disk confocal microscope. Note that the beads are less porous than those in panel **a**, so binding appears as a bright ring around the bead perimeter. Scale bar equals 100 µm.

AlphaFold predicts that the KPWE motif binds specifically to the bottom of the PEX7 β-propeller (Fig. 3c and Extended Data Fig. 4 for the associated pLDDT confidence scores of all AlphaFold models). The interface is highly conserved (Fig. 3d) and shows that each of the amino acids comprising the KPWE motif engages in a unique interaction with PEX7: the lysine projects into the acidic core of the β-propeller, the proline is nestled in a hydrophobic pocket, the tryptophan is sandwiched between the aliphatic stem of a conserved lysine in PEX7 and the lysine from the KPWE motif, and the glutamate forms polar contacts with a conserved arginine in PEX7 (Extended Data Fig. 5). The interaction with the KPWE motif notably occurs on the side of the PEX7 β-propeller opposite to where the PTS2 signal and import receptors bind (Fig. 3e). The two interfaces are indeed independent, as beads conjugated to the KPWE peptide could recruit PTS2–GFP only when PEX7 and the receptor were both present (Extended Data Fig. 6a). No binding was seen in the absence of PEX7 or with beads harbouring the scrambled peptide. These results imply that PEX7 might enter the YG phase either by binding directly to the KPWE motif or by piggybacking on an import receptor in the presence of cargo.

Direct entry of PEX7 into the YG phase could be demonstrated with the hydrogel system. Using hydrogels prepared from a recombinant fragment of PEX13 consisting of the wild-type unstructured region and YG domain (Fig. 3f), we observed that PEX7–GFP readily entered the phase, whereas GFP alone did not (Fig. 3g). Ablation of the KPWE motif did not affect gelation (Fig. 3f) but it precluded the entry of PEX7–GFP (Fig. 3g). By contrast, PEX5 fused at the C terminus to GFP (PEX5–GFP) accumulated equally well in either wild-type or mutant hydrogels (Fig. 3g). Thus, PEX7 interacts with the YG phase through the KPWE motif instead of through the YG repeats to which import receptors bind.

We next examined how PEX7 interacts with the YG phase when bound to a receptor and cargo. We prepared a complex between PEX7–GFP and the chimaeric receptor PEX5-21 in the presence of a synthetic peptide comprising the PTS2 signal. Formation of the complex was verified using beads coated with the receptor, which could recruit PEX7–GFP only in the presence of the PTS2 peptide (Extended Data Fig. 6b). The complex could partition into the YG hydrogel even when the KPWE motif was absent (Fig. 3g), revealing that the receptor is sufficient to drag PEX7 and cargo into the YG phase. This result was recapitulated using a complex consisting of PEX7 bound to the receptor and PTS2–GFP cargo, which readily entered the gel regardless of the KPWE motif (Fig. 3g).

Together, the hydrogel experiments demonstrate that PEX7 and import receptors can partition into the YG phase independently of each other. When both components assemble with cargo into a complex, each of these interactions may be utilized to move the cargo through the phase into peroxisomes. Interestingly, PEX5–GFP permeated further into the wild-type hydrogel than either PEX7–GFP alone or PEX7–GFP in the cargo complex (Fig. 3g). Thus, PEX7 seems to traverse the YG phase more slowly, consistent with PTS2 cargo being moved through the phase slower than PTS1 cargo (Fig. 2c). Efficient translocation into peroxisomes might therefore primarily be driven by the receptor.

### The KPWE motif mediates PEX7 recycling

**Fig. 4:**
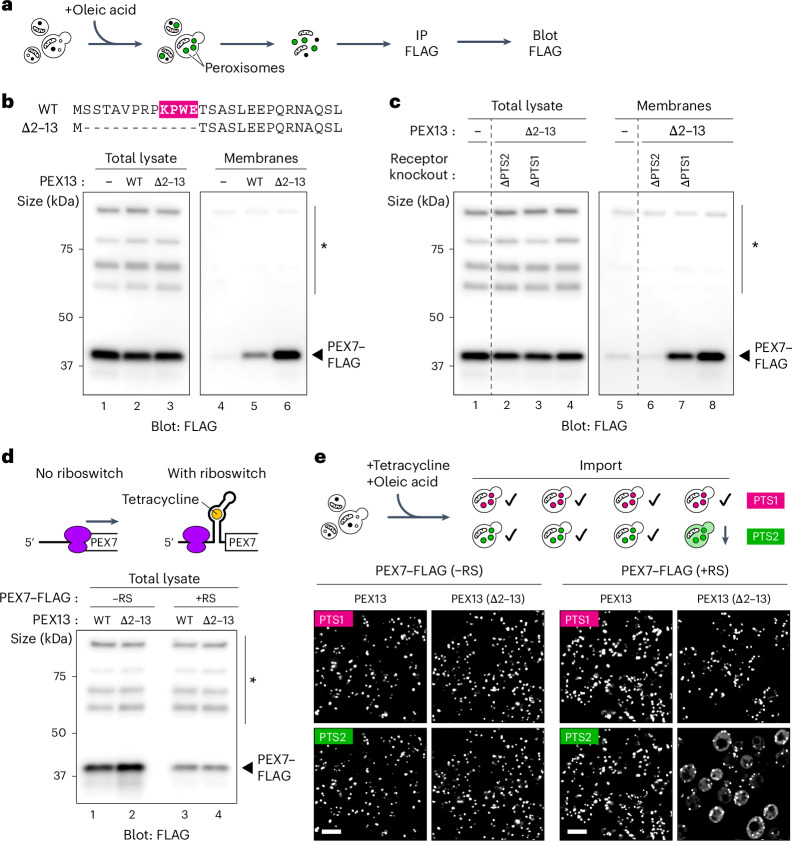
PEX7 recycling requires the KPWE motif of PEX13. **a**, PEX7 fused at the C terminus to a FLAG epitope tag (PEX7–FLAG) was expressed in yeast strains containing different PEX13 mutants. The cells were cultured in oleic acid to induce peroxisomes, lysed and then separated into a peroxisome-containing membrane fraction. PEX7–FLAG was immunoprecipitated (IP) from the lysate and the detergent-solubilized membranes, resolved by SDS–PAGE and detected by immunoblotting for the FLAG epitope. **b**, PEX7–FLAG was analysed as in **a** for cells lacking PEX13 (−), or cells containing either wild-type PEX13 (WT) or a PEX13 mutant lacking residues 2–13 (∆2–13) that encompass the KPWE motif, as shown (top). Immunoblots of total lysates and corresponding membrane fractions (bottom). **c**, As in **b** but including strains additionally lacking the PTS2 receptors PEX18 and PEX21 (∆PTS2) or the PTS1 receptors PEX5 and PEX9 (∆PTS1). **d**, PEX7 expression was decreased by inserting a tetracycline-responsive riboswitch upstream of the coding sequence (top). Total lysates from cells expressing PEX7–FLAG with or without the riboswitch were analysed by immunoblotting as in **a** (bottom). **e**, Peroxisomes were induced in cells expressing PEX7 (with or without the riboswitch as indicated) as well as either wild-type PEX13 or PEX13 lacking the KPWE motif (∆2–13). Protein import was assessed by fluorescence microscopy using endogenously expressed FOX2 fused to RFP (PTS1) and POT1 fused to GFP (PTS2). Bright puncta indicate successful import, whereas the absence of import manifests as a diffuse signal throughout the cytosol. Scale bars, 5 µm. *Non-specific bands; RS, riboswitch. Unprocessed blots are provided. Source data

**Extended Data Fig. 7:**
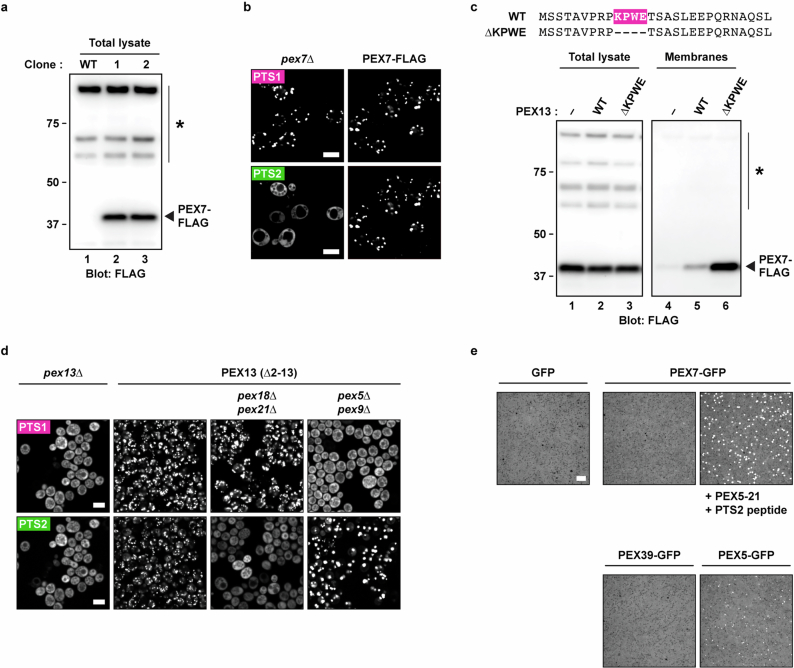
Epitope-tagging PEX7 in yeast and functional characterization. **a**, PEX7 was fused at the C terminus to a FLAG tag (PEX7–FLAG) and expressed at endogenous levels in yeast. Shown is a FLAG immunoblot of the total lysate from cells expressing wild-type (WT) untagged PEX7 or two clones expressing PEX7–FLAG. Asterisk indicates non-specific bands; molecular weights (in kD) are marked on the left. **b**, Yeast cells lacking PEX7 (*pex7*∆), or expressing PEX7–FLAG, were grown in oleic acid to induce peroxisomes. Peroxisomal protein import was assessed by imaging the endogenous matrix proteins FOX2 fused to RFP (PTS1) and POT1 fused to GFP (PTS2). Note that bright puncta indicate successful import; in the absence of import, the cargo is diffusely distributed throughout the cytosol. Scale bar equals 5 µm. **c**, PEX7–FLAG was expressed in cells lacking PEX13 (*pex13*∆), or cells containing wild-type (WT) PEX13 or a mutant lacking the KPWE motif (∆KPWE). Cells were grown in oleic acid to induce peroxisomes, then lysed and separated into a peroxisome-containing membrane fraction. PEX7–FLAG was immunoprecipitated from the total lysate and the corresponding detergent-solubilized membranes, resolved by SDS–PAGE, and detected by immunoblotting for the FLAG epitope. Asterisk marks non-specific bands; molecular weights (in kD) are marked on the left. **d**, Yeast cells devoid of PEX13 (*pex13*∆), or expressing PEX13 lacking residues 2–13 that encompass the KPWE motif, were grown in oleic acid to induce peroxisomes. Where indicated, the cells additionally lacked the two PTS2 receptors PEX18 and PEX21 (*pex18*∆ *pex21*∆) or the two PTS1 receptors PEX5 and PEX9 (*pex5*∆ *pex9*∆). Peroxisomal protein import was assessed as in panel **b**. Scale bar equals 5 µm. **e**, *Xenopus* egg extract was incubated for 1 h with 0.5 µM GFP alone or GFP fused to the C terminus of the indicated proteins, then imaged on a spinning-disk confocal microscope. Where indicated, reactions additionally included the chimeric receptor PEX5-21 (described in Fig. 1b) at 0.5 µM and a synthetic peptide comprising the PTS2 signal from yeast POT1. Note that puncta reveal entry of the fluorescent species into peroxisomes. Scale bar equals 5 µm. Unprocessed blots are available. Source data

We wondered whether the interaction between PEX7 and the KPWE motif allows PEX7 to return to the cytosol after PEX7 has dissociated from the receptor and cargo inside the matrix. To test this hypothesis, we ablated the KPWE motif in PEX13 in yeast and investigated whether PEX7 would accumulate in peroxisomes. To enable detection of PEX7 by immunoblotting, we introduced a single FLAG tag at the C terminus of the protein (Extended Data Fig. 7a). The tagged form was expressed from the endogenous locus and was fully functional, as assessed in cells expressing fluorescently labelled PTS1 and PTS2 matrix proteins (Extended Data Fig. 7b). The corresponding strains were cultured in oleic acid to induce peroxisomes and the cells subsequently fractionated into a peroxisome-containing membrane pellet (Fig. 4a). PEX7–FLAG was indeed considerably enriched in the membrane fraction in the absence of the KPWE motif (Fig. 4b and Extended Data Fig. 7c). Given that PEX7 completely enters the peroxisomal matrix during import^28–35^, this observation suggests that PEX7 cannot return to the cytosol when its interaction with the YG phase is disrupted. Consistent with this assumption, PEX7–FLAG accumulation was abolished after deletion of the two yeast PTS2 receptors PEX18 and PEX21 (Fig. 4c). By contrast, deletion of the two PTS1 receptors PEX5 and PEX9 had only a small effect (Fig. 4c). All receptor-pair knockouts correctly precluded import of their respective cargo (Extended Data Fig. 7d).

Experiments in *Xenopus* egg extract confirmed that PEX7–GFP enters peroxisomes only when it is assembled into a complex with PEX5 and cargo (Extended Data Fig. 7e). Interestingly, PEX7–GFP puncta were considerably brighter than those formed by PEX5–GFP (Extended Data Fig. 7e), consistent with the hypothesis that the two proteins are not recycled together. This conclusion agrees with a previous study^35^ showing that PEX7 is recycled more slowly than PEX5. Together, our results suggest that PEX7 enters peroxisomes along with cargo by piggybacking on an import receptor; PEX7 then returns to the cytosol by binding to the KPWE motif of PEX13 and thereby partitioning back into the YG phase from inside the matrix.

The proposed model implies that KPWE-motif deletion should inhibit PTS2 import because PEX7 should no longer be recycled. Surprisingly, however, cells lacking the KPWE motif showed no obvious PTS2 import defect (Extended Data Fig. 7d). We reasoned that in our assay, PEX7 might be sufficiently abundant to mask a recycling phenotype. We therefore inserted a tetracycline-responsive riboswitch^39^ upstream of the PEX7 open reading frame to reduce translation of the corresponding PEX7 messenger RNA. This strategy decreased the abundance of PEX7 by about an order of magnitude (Fig. 4d). Under these conditions, PTS2 import was still normal in cells that retained the KPWE motif, whereas cells that lacked the KPWE motif now had a pronounced import defect specific to PTS2 proteins (Fig. 4e). These results confirm the proposed role of the KPWE motif in PEX7 recycling and further demonstrate that PEX7 recycling is essential for protein import when PEX7 becomes limiting in cells.

### PEX7 release from the YG phase into the cytosol

**Fig. 5:**
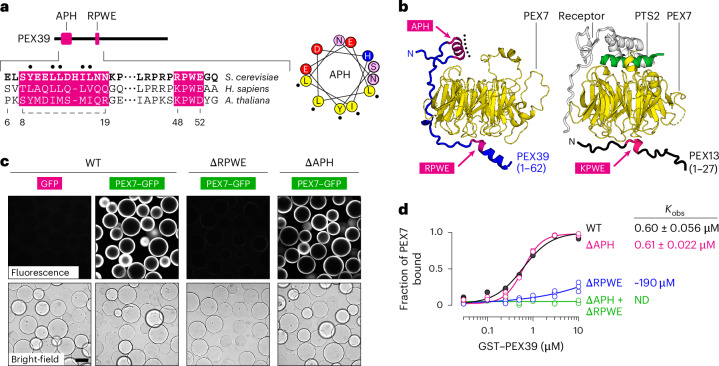
PEX39 binds to PEX7 using a KPWE-like motif. **a**, Location of the APH and KPWE-like motif (RPWE) in yeast PEX39 with corresponding amino-acid sequences from the indicated species (UniProt ID: P47087, Q5I0X4 and A0A5S9YGZ4). Numbered residues correspond to the yeast homologue. Conserved hydrophobic residues in the APH are marked with back dots in the helical-wheel diagram. **b**, AlphaFold model of the complex between yeast PEX7 and the region of yeast PEX39 that includes the APH and RPWE motif (left). The hydrophobic surface of the APH is marked by black dots. Model of the region of yeast PEX13 that includes the KPWE motif overlaid on the crystal structure of yeast PEX7, the PTS2 signal and the yeast PTS2 receptor PEX21 (PDB: 3W15; right). **c**, Glutathione-conjugated beads were bound to glutathione-*S*-transferase (GST)-tagged wild-type yeast PEX39 (WT), or mutants whose RPWE motif (∆RPWE) or conserved hydrophobic residues in the APH (∆APH) were converted to alanine. Recruitment of yeast PEX7–GFP to the beads after 1 h was visualized on a spinning-disk confocal microscope. Scale bar, 100 µm. **d**, Glutathione-conjugated beads were incubated with 1 µM yeast PEX7 and varying concentrations of the indicated GST-tagged forms of yeast PEX39. The beads and any bound protein were sedimented after 1 h. Each supernatant fraction was resolved by SDS–PAGE and stained with Coomassie blue (representative images of the gels in Extended Data Fig. 9b). PEX7 remaining in the supernatant was quantified and used to calculate the bound fraction, which was plotted as a function of the PEX39 concentration. Lines represent fits, generated by least-squares regression, to the data from three independent experiments; the corresponding observed binding constants (*K*_obs_) are indicated (right; mean ± s.e.m.). ND, binding not detectable. Source numerical data are provided. Source data

**Extended Data Fig. 8:**
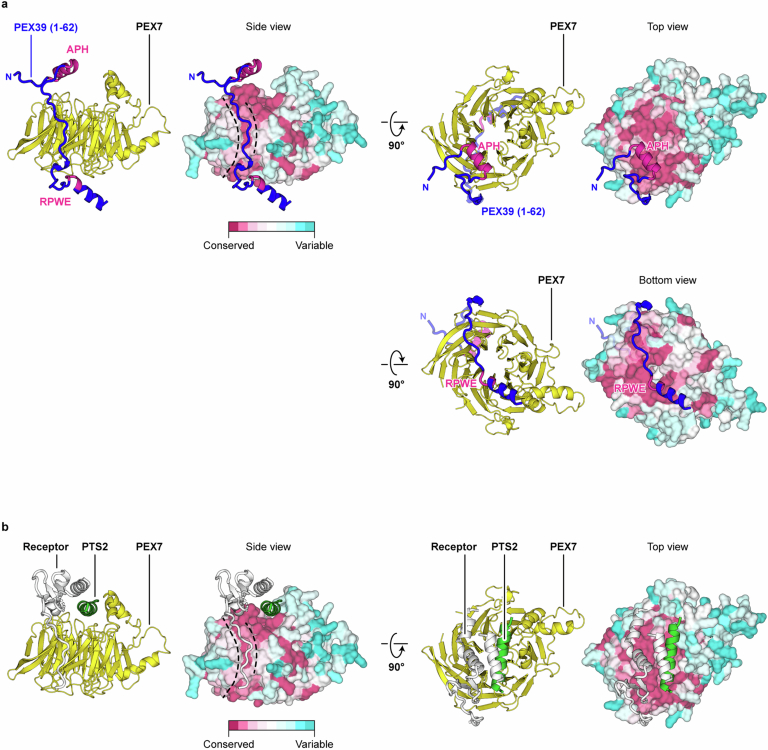
Conservation of PEX39-binding sites on PEX7. **a**, The leftmost panel shows an AlphaFold model of the complex between yeast PEX7 and the indicated region of yeast PEX39 containing the APH and the RPWE motif. The N terminus of PEX39 is marked for reference. The next panel on the right shows the same complex but with PEX7 represented as a surface and coloured according to the amino-acid conservation scale underneath. A conserved groove along the side of PEX7 is framed by dashed black lines. The four rightmost panels show the same complex but rotated as indicated. Note the extensive amino-acid conservation around the binding sites for the APH and the RPWE motif. **b**, As in panel **a**, showing the crystal structure (PDB: 3W15) of the complex between yeast PEX7, a PTS2 peptide, and the yeast PTS2 receptor PEX21. The complex is overlaid on the AlphaFold model of yeast PEX7 shown in panel **a** (with PEX7 from the crystal structure not visible). Note that a portion of the receptor snakes along the same conserved groove (framed by dashed black lines) on the side of PEX7 to which PEX39 is predicted to bind. Furthermore, the receptor and PTS2 peptide are cradled by the same conserved surface on top of PEX7 that also binds to the APH of PEX39.

Given the favourable interaction between PEX7 and the YG phase, we next investigated how PEX7 would be extracted from the phase and returned to the cytosol to start another import cycle. A possible mechanism was suggested by large-scale protein-interaction studies in yeast^40,41^ and mammalian cells^42–44^, which had identified an uncharacterized binding partner of PEX7 in the cytosol that has recently been named PEX39 (ref. ^45^). The protein is conserved throughout eukaryotes and contains an amino-acid sequence (RPWE in yeast) that resembles the KPWE motif of PEX13 (Fig. 5a). PEX39 also has a conserved amphipathic helix (APH) near its N terminus (Fig. 5a). AlphaFold predicts that PEX39 can bind to PEX7 using both of these features: the RPWE motif binds to the same site as the analogous motif of PEX13 and the APH binds to the opposite side, where the PTS2 signal and import receptors are recruited (Fig. 5b). Both interaction surfaces on PEX7 are conserved in addition to a groove that cradles the linker between the RPWE motif and the APH (Extended Data Fig. 8).

**Extended Data Fig. 9:**
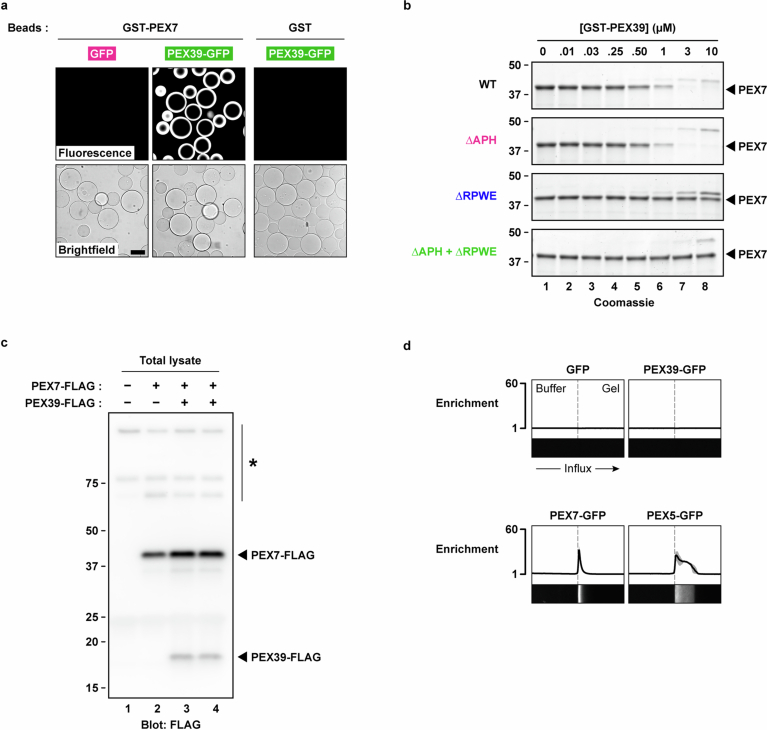
Characterization of the interaction between PEX39 and PEX7, the abundance of PEX39 in yeast, and the interaction of PEX39 with the YG phase. **a**, Glutathione-conjugated beads were bound to 1 µM purified recombinant GST-tagged yeast PEX7 or GST alone, and incubated with 1 µM purified recombinant yeast PEX39 fused at the C terminus to GFP (PEX39–GFP) or with GFP alone. Recruitment of each fluorescent protein to the beads was visualized after 1 h on a spinning-disk confocal microscope. Scale bar equals 100 µm. **b**, Representative Coomassie-stained SDS–PAGE gels corresponding to the binding experiment in Fig. 5d. Briefly, yeast PEX7 was incubated with beads coated with GST-tagged wild-type yeast PEX39 or the indicated mutants; after sedimenting the beads, each supernatant fraction was resolved by SDS–PAGE and stained by Coomassie Blue. Molecular weights (in kD) are marked on the left. **c**, PEX7 and PEX39 were fused at their C terminus to a FLAG tag (PEX7–FLAG and PEX39–FLAG) and expressed from their respective endogenous promoters in yeast. Shown is a FLAG immunoblot of the total lysate from two different clones (lanes 3 and 4) following growth in oleic acid, compared to cells expressing PEX7–FLAG and untagged PEX39 (lane 2), and cells expressing untagged versions of both proteins (lane 1). Asterisk indicates non-specific bands; molecular weights (in kD) are marked on the left. **d**, YG hydrogels were incubated with GFP alone or with GFP fused to the C terminus of the indicated proteins. The accumulation of each fluorescent protein inside the gel after 30 min was visualized on a point-scanning confocal microscope. Representative images are shown; the fold enrichment of each protein relative to buffer, across the imaged field, is plotted above (mean ± range of 3 experiments). The width of each image corresponds to 300 µm. Source numerical data and unprocessed blots are provided. Source data

The predicted interactions were validated by showing that beads coated with PEX7 could recruit PEX39 fused at the C terminus to GFP (PEX39–GFP; Extended Data Fig. 9a). Conversely, beads coated with PEX39 could recruit PEX7–GFP and the recruitment required the RPWE motif (Fig. 5c). Mutations in the APH resulted in marginally reduced binding. These results were quantitatively confirmed by incubating PEX7 with varying concentrations of PEX39 immobilized on beads (Extended Data Fig. 9b). Measurements of the amount of PEX7 remaining in solution revealed similar binding constants for wild-type PEX39 and the mutant lacking the APH, whereas the mutant lacking the RPWE motif bound considerably weaker (Fig. 5d). No binding was measurable in the absence of both the RPWE motif and the APH. Thus, PEX39 can directly bind to PEX7, with the RPWE motif driving the interaction.

**Fig. 6:**
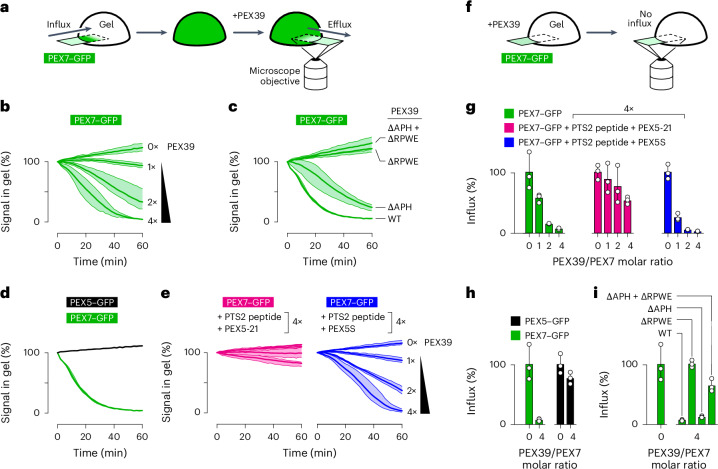
PEX39 can extract PEX7 from the YG phase. **a**, YG hydrogels were prepared in a glass-bottomed dish and pre-equilibrated with PEX7–GFP. PEX39 was then added to the buffer and a point-scanning confocal microscope was used to follow the PEX7–GFP fluorescence over time. **b**, PEX7–GFP signal in the gel over time at the indicated molar ratios of wild-type PEX39 (relative to PEX7). The mean (bold line) and range (shading) of three experiments are shown. **c**, As in **b** using a fourfold excess of wild-type (WT) PEX39 or mutants harbouring alanines in place of the APH (∆APH), the RPWE motif (∆RPWE) or both (∆APH + ∆RPWE). **d**, As in **b** using PEX7–GFP or PEX5–GFP and a fourfold excess of wild-type PEX39. **e**, As in **b** using a fourfold excess of PTS2 peptide and the PEX7-binding chimaeric receptor PEX5-21 (left) or PTS2 peptide and the non-PEX7-binding receptor isoform PEX5S (right). Wild-type PEX39 was titrated as indicated. **f**, YG hydrogel droplets were incubated with PEX7–GFP in the presence of PEX39. The effect of PEX39 on the influx of PEX7–GFP into the gel was then assessed using a point-scanning confocal microscope. **g**, PEX7–GFP influx in the presence of the indicated molar ratios of wild-type PEX39 (relative to PEX7) with or without a fourfold excess of PTS2 peptide and either PEX5S or PEX5-21. The mean (bars) and individual values (circles) of three experiments are shown. **h**, As in **g** using PEX7–GFP or PEX5–GFP and the indicated molar ratios of wild-type PEX39. **i**, As in **g** using wild-type PEX39 or the indicated mutants. Source numerical data are provided. Source data

Because PEX39 and PEX13 can each bind to PEX7 using similar motifs, we investigated whether PEX39 could extract PEX7 from the YG phase. We tested this hypothesis by preloading YG hydrogels with PEX7–GFP, then spiking the buffer with increasing concentrations of PEX39 and following the PEX7–GFP fluorescence in the gel over time (Fig. 6a). Addition of PEX39 rapidly depleted PEX7–GFP from the hydrogel in a dose-dependent manner, whereas PEX7–GFP continued to accumulate in the gel in the absence of PEX39 (Fig. 6b). The extraction of PEX7–GFP was abolished by mutating the RPWE motif in PEX39 (Fig. 6c), whereas mutating the APH had a much smaller effect, consistent with our binding experiments. Ablation of both motifs had no additional effect. PEX39 was notably unable to extract PEX5–GFP from the hydrogel (Fig. 6d). These results thus support the hypothesis that PEX39 can extract PEX7 from the YG phase during recycling, when PEX7 would no longer be bound to cargo and an import receptor.

The proposed role of PEX39 in PEX7 recycling implies that PEX39 should not extract PEX7 from the YG phase during translocation into peroxisomes when PEX7 would be loaded with cargo and an import receptor. PEX39 was indeed unable to efficiently extract PEX7–GFP from YG hydrogels in the presence of a PTS2 peptide and the chimaeric receptor PEX5-21, even when PEX39 and the receptor were both present in excess molar ratios relative to PEX7–GFP (Fig. 6e). By contrast, PEX7–GFP was readily extracted by PEX39 in the presence of the short receptor isoform PEX5S that cannot bind to PEX7 (Fig. 6e). Thus, PEX39 does not interfere with the translocation of the fully assembled PEX7–receptor–cargo complex into peroxisomes.

To confirm this conclusion, we monitored the influx of PEX7–GFP into the hydrogel in the presence of PEX39 (Fig. 6f). As expected, PEX39 blocked the influx of PEX7–GFP alone as well as PEX7–GFP in the presence of a PTS2 peptide and PEX5S (Fig. 6g). By contrast, PEX39 was much less inhibitory when PEX7–GFP was assembled into a complex with the PTS2 peptide and the chimaeric receptor PEX5-21 (Fig. 6g). Some inhibition was observed at high PEX39 concentrations, suggesting that PEX39 cannot be too abundant in vivo; PEX39 is indeed a few-fold less abundant than PEX7 in yeast (Extended Data Fig. 9c). Consistent with the hydrogel release experiments, PEX39 did not prevent the influx of PEX5–GFP (Fig. 6h), and the observed inhibition of PEX7–GFP influx depended on the RPWE motif and much less on the APH (Fig. 6i). PEX39–GFP did not by itself enter the YG hydrogel (Extended Data Fig. 9d) and was not imported into peroxisomes in *Xenopus* egg extract (Extended Data Fig. 7e).

Together, our results lead to a model in which import receptors move cargo-loaded PEX7 through the YG conduit into peroxisomes. After dissociating from the receptor and cargo inside the matrix, PEX7 moves back on its own through the YG conduit and is extracted out of the YG phase into the cytosol by PEX39.

### Reloading PEX7 with cargo

**Fig. 7:**
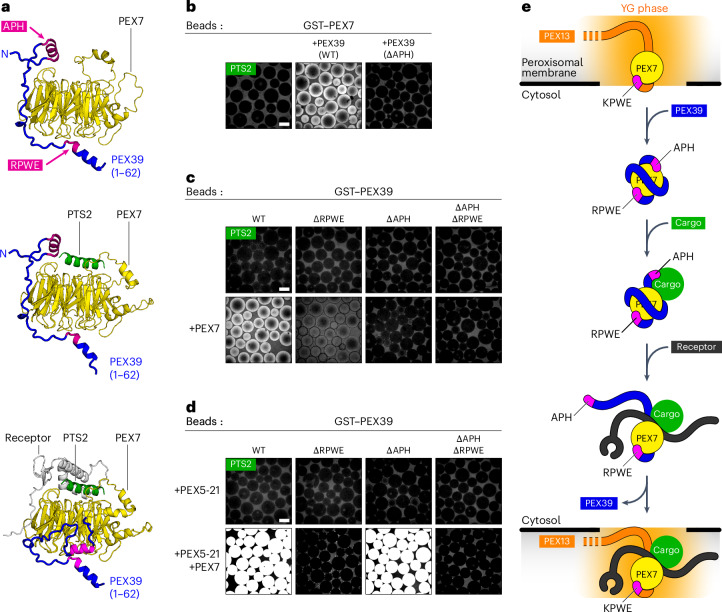
PEX39 promotes cargo binding to PEX7. **a**, AlphaFold models of the complex between yeast PEX7 and the region of yeast PEX39 that includes the APH and RPWE motif, with and without a PTS2 signal and the yeast PTS2 receptor PEX21. Note that PEX7 alone binds to both the APH and RPWE motif (top). The APH additionally stabilizes the interaction with the PTS2 signal (middle), whereas binding of the receptor displaces the APH (bottom). The RPWE motif remains bound in all cases. **b**, Glutathione-conjugated beads were incubated overnight with 5 µM GST-tagged yeast PEX7 (GST–PEX7) and 1 µM GFP fused at the N terminus to the PTS2 signal from yeast POT1 (PTS2–GFP). Where indicated, reactions included 5 µM wild-type (WT) yeast PEX39 or a mutant whose conserved hydrophobic residues in the APH were converted to alanine (∆APH). Recruitment of PTS2–GFP to the beads was visualized on a spinning-disk confocal microscope. **c**, As in **b** using GST-tagged wild-type yeast PEX39 (GST–PEX39) or the indicated GST-tagged mutants and yeast PEX7 at 5 µM. **d**, As in **c** with the additional presence of the chimaeric receptor PEX5-21 (5 µM). Contrast was adjusted as in **b**. **e**, Proposed steps for reloading of PEX7 with receptor and cargo. Scale bars, 100 µm.

To start another round of translocation, PEX7 has to be reloaded with cargo and a new receptor molecule. AlphaFold predicts that the APH of PEX39 can stabilize the interaction between PEX7 and the PTS2 signal (Fig. 7a), suggesting that PEX39 might promote cargo binding. PTS2–GFP cargo was indeed recruited to PEX7-coated beads in the presence of PEX39 (Fig. 7b) and conversely, PTS2–GFP was recruited to PEX39-coated beads in the presence of PEX7 (Fig. 7c). The cargo did not bind to either component alone. APH mutation abolished cargo binding to the PEX39–PEX7 complex (Fig. 7b,c), consistent with the APH stabilizing the interaction with the PTS2 signal. Notably, cargo binding was also reduced following mutation of the RPWE motif, as expected from the role of the motif in driving the interaction with PEX7. Thus, PEX39 not only extracts PEX7 from the YG phase but also enables PEX7 to rebind to cargo (Fig. 7e).

### Dissociation of PEX39 from cargo-loaded PEX7

Dissociation of PEX39 would have to occur in two steps. First, an import receptor would displace the APH of PEX39, as predicted by AlphaFold (Fig. 7a). The prediction is supported by experiments in which a complex between PEX7, the chimaeric receptor PEX5-21 and PTS2–GFP cargo could be recruited to beads coated with wild-type PEX39 (Fig. 7d). Recruitment was abolished by mutation of the PEX39 RPWE motif and completely unaffected by mutation of the APH (Fig. 7d). Thus, after receptor binding, PEX39 only interacts with PEX7 through its RPWE motif (Fig. 7e).

**Fig. 8:**
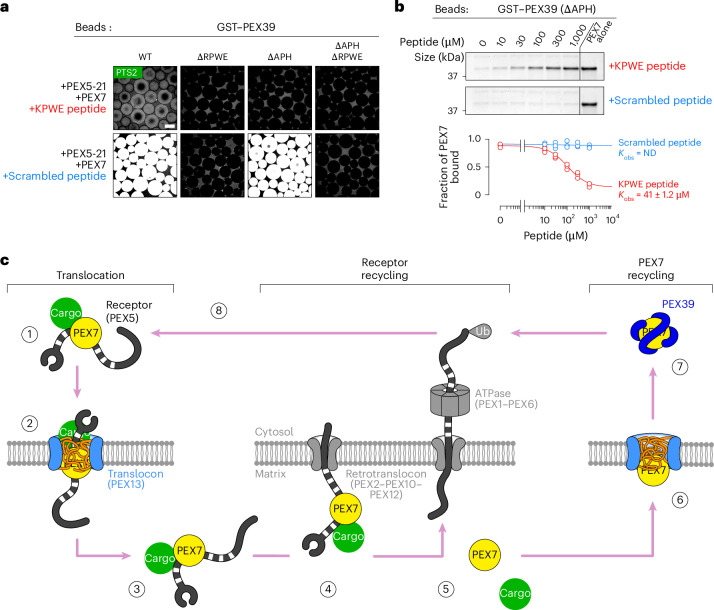
Dissociation of PEX39 from cargo-loaded PEX7 and overall model of PTS2 protein import. **a**, Glutathione-conjugated beads were incubated overnight with 5 µM GST-tagged wild-type yeast PEX39 or the indicated mutants in the presence of 5 µM PEX5-21 and yeast PEX7 as well as 1 µM PTS2–GFP. Where indicated, reactions included 100 µM of a peptide comprising the KPWE motif of PEX13 or a scrambled version. Recruitment of PTS2–GFP to the beads was visualized on a spinning-disk confocal microscope. Scale bar, 100 µm. **b**, Glutathione-conjugated beads were first incubated with yeast PEX7 and the ∆APH mutant of GST-tagged PEX39 (each at 1 µM), and then with varying concentrations of the KPWE or scrambled peptide. The beads were sedimented and the supernatant fraction was resolved by SDS–PAGE, followed by Coomassie blue staining. Representative gels are shown (top), with relative sizes marked in kDa on the left. Fraction of PEX7 remaining on the beads in three independent experiments plotted against the peptide concentration and fitted to a competitive-binding isotherm (bottom). The mean ± s.e.m. observed binding constant (*K*_obs_) for each peptide is shown; ND, binding not detectable. **c**, Model of PTS2 protein import into peroxisomes. Unprocessed gels and source numerical data are provided. Source data

Finally, complete dissociation of PEX39 would occur following re-entry of the PEX7–receptor–cargo complex into the YG conduit, where the KPWE motif of PEX13 would displace the RPWE motif of PEX39. The PEX7–receptor–cargo complex was indeed no longer efficiently recruited to PEX39-coated beads in the presence of the KPWE peptide, whereas the scrambled peptide had no effect (Fig. 8a). Furthermore, the KPWE peptide could strip off PEX7 from PEX39 lacking the APH, whereas the scrambled peptide could not (Fig. 8b). Together, our results reveal that PEX39 not only extracts PEX7 from the YG conduit but also allows PEX7 to rebind to cargo. The subsequent recruitment of an import receptor partially displaces PEX39 and allows the cargo complex to partition back into the YG conduit. As the receptor drags the complex into the YG phase, the presence of multiple KPWE motifs of PEX13 effectively displaces the RPWE motif of PEX39, thereby releasing PEX39 into the cytosol (Fig. 7e).

## Discussion

Using a combination of in vitro reconstituted systems and experiments in vivo, we have determined the mechanism by which PTS2 matrix proteins are imported into peroxisomes (Fig. 8c). Translocation is initiated by the assembly of PTS2 cargo into a complex with PEX7 and an import receptor in the cytosol (step 1). The receptor can either be the long isoform of PEX5 in higher organisms or one of its paralogues such as PEX18 or PEX21 in yeast. The cargo complex then partitions into a conduit that consists of a selective phase formed from the YG domain and the preceding unstructured region of PEX13 (step 2). Translocation through the phase is mediated by interactions between the WXXXF/Y motifs of the receptor and the YG repeats of PEX13 as well as by the interaction between PEX7 and the KPWE motif of PEX13. These interactions allow the cargo complex to move across the membrane into the organelle (step 3). Receptor recycling is initiated by the insertion of the receptor’s flexible N terminus into the pore of the PEX2–PEX10–PEX12 ubiquitin ligase complex from within the matrix (step 4). Mono-ubiquitination of the receptor’s N-terminal cysteine then recruits the PEX1–PEX6 ATPase, which pulls the receptor through the ligase pore and causes the cargo as well as PEX7 to be stripped off and left behind inside the matrix (step 5). After being released from the receptor, PEX7 repartitions into the YG phase via the interaction with the KPWE motif and thereby diffuses back across the membrane (step 6). PEX7 is extracted out of the phase on the cytosolic side by PEX39 (step 7), which can then facilitate the reloading of PEX7 with cargo and a new receptor molecule to start another import cycle (step 8). The subsequent re-entry of the cargo complex into the YG conduit releases PEX39 and leaves it behind in the cytosol.

During translocation into peroxisomes, both PEX7 and import receptors can interact with the YG phase independently of each other and probably cooperate to move cargo through the import conduit. However, receptors move through YG hydrogels faster than PEX7, suggesting that translocation is predominantly driven by the receptors. PEX7 instead seems to strengthen the interaction of the cargo complex with the YG phase, causing the PTS2–cargo complex to move through the phase more slowly. This observation probably explains why PTS2 import in *Xenopus* egg extract is slower than import of PTS1 cargo, which has also been reported in plants^46^.

Because diffusion through the YG phase is non-directional, translocation must be biased towards the peroxisomal matrix. Directionality is exclusively imposed by the receptors, which can bind with high affinity to the luminal domain of PEX14 (a membrane protein closely associated with PEX13) using their WXXXF/Y motifs^47^. This interaction would attract the cargo complex into the matrix and would explain why the WXXXF/Y motifs of the receptors are essential for PTS2 import in *Xenopus* egg extract but not for permeation of the PTS2–cargo complex through the YG hydrogel. This model also agrees with the observation that the recombinant luminal domain of PEX14 blocks PTS2 import in rat-liver extract^35^. Translocation could additionally be driven inwards if import were coupled to receptor export^48^. The N terminus of the receptor could insert into the pore of the ubiquitin ligase complex and recruit the PEX1–PEX6 ATPase before the remainder of the cargo complex has moved through the import conduit; the ATPase would then pull the receptor together with bound PEX7 and cargo through the YG phase into the organelle, as suggested previously^49^. Although this mechanism might not be needed for import in every case^35,50^, it might expedite the translocation of bulkier or more YG-phobic cargo whose passage through the YG phase might be unfavourable. Regardless of the mechanism, the biased movement of the receptors would result in import of both PTS1 and PTS2 cargo, and would also explain how PEX7 enters the matrix.

Our work reveals that import receptors and PEX7 return to the cytosol by distinct mechanisms. Import receptors are actively pulled out through a retrotranslocon (the ubiquitin ligase complex) by a process powered by nucleotide hydrolysis^21^. By contrast, PEX7 recycling is driven solely by protein interactions with the YG phase and PEX39. The existence of separate recycling pathways is supported by experiments in rat-liver extract, which have shown that PEX7 returns to the cytosol slower than import receptors^35^; this difference is likely to reflect the slow permeation of PEX7 through the YG phase revealed by our hydrogel system. Nonetheless, PEX7 recycling depends on the extraction of the receptor from the organelle^35,51^, consistent with our model that PEX7 must be stripped from the receptor before re-entering the YG conduit.

The role of PEX39 as a chaperone for PEX7 is supported by a recent preprint^45^. Although this study did not investigate PEX39 in the context of PEX7 recycling, it likewise reports that PEX39 functions in PTS2 import, stabilizes the interaction between PEX7 and cargo, and binds to PEX7 more strongly than PEX13. These observations are in accord with our model in which PEX39 extracts PEX7 from the YG phase and then enables PEX7 to rebind to cargo and re-enter the phase during the next import cycle. Our model implies that PEX39 functions near the membrane, which is consistent with the observation that the PEX39 homologue in plants is fused to the cytosolic C terminus of the PEX14 membrane protein^45^. The authors additionally report that PTS2 import is attenuated by both downregulation and overexpression of PEX39, which is again consistent with our model: some PEX39 is required to recycle PEX7 but excess PEX39 would prevent PEX7 from binding to cargo and receptors, and would also inhibit the entry of the cargo complex into the YG phase. PEX39 does not need to be very abundant because PEX7 recycling is required only when PEX7 becomes limiting; a basal recycling rate coupled with new protein synthesis is probably sufficient to maintain a stable cytosolic pool of PEX7 under physiological conditions. Recycling might be critical under disease conditions where PEX7 expression is attenuated by hypomorphic alleles or destabilizing mutations. Boosting this recycling pathway might be an attractive therapeutic strategy to restore PTS2 import in such contexts.

The bidirectional translocation of PEX7 through the YG phase resembles the passage of nuclear transport receptors into and out of the nucleus through nuclear pores. In fact, PEX7 is uncannily similar to the mRNA-export factor GLE2 (also known as RAE1 in mammals). Both proteins consist of WD40 repeats^24,52^ and bind to short linear motifs in phase-forming polypeptides: PEX7 to the KPWE sequence in the unstructured region of PEX13 and GLE2 to the GLE2-binding sequence (GLEBS) in the unstructured FG nucleoporin NUP116 (also known as NUP98 in mammals)^53^. Akin to nuclear transport receptors and their intrinsic affinity for the nucleoporin FG phase^19^, PEX7 also seems to have weak affinity for the peroxisomal YG phase because some entry of PEX7 into YG hydrogels was observed in the absence of the KPWE motif. The similarity between PEX7 and nuclear transport receptors therefore reinforces the analogy between peroxisomal matrix-protein import and nuclear-protein transport.

The analogy implies that the two protein-transport systems probably evolved from the same ancestral machinery, which might have utilized a selective phase to move folded proteins between compartments. The most primitive systems might have allowed bidirectional transport of receptor–cargo complexes through a conduit containing the phase without directional bias. In the case of peroxisomal-protein import, this system might have consisted of PEX7, a PTS2 signal and a PEX13-like pore. Some directionality might have been provided by the removal of the PTS2 signal by a matrix protease, which still occurs in some organisms^7^. PEX5 and the PTS1 signal would have evolved later to provide directional bias and enhance the efficiency of translocation. Supporting this theory, only a few PTS2 proteins occur in most contemporary organisms yet include some of the most conserved and arguably ancient peroxisomal enzymes such as thiolase; furthermore, in certain species, PTS2 import has been completely supplanted by the PTS1 system^7^.

## Methods

All reagents were obtained from Millipore Sigma unless specified otherwise. Buffers were prepared in ultrapure water. Room temperature (RT) indicates 22–25 °C.

### Plasmid construction

#### Plasmids for recombinant-protein expression in *Escherichia coli*

Plasmids were derived from vector pET-28b(+) (Novagen) and are listed in Supplementary Table 1 along with details about their construction and relevant GenBank accession numbers. All constructs were codon-optimized for *E. coli* and verified by whole-plasmid sequencing. The construct for expressing the N-terminal unstructured region (including the YG domain) of PEX13 was described previously^17^. For constructs fused to GST, the GST coding sequence was first amplified by PCR from vector pGEX-6P-3 (Cytiva), incorporating at the 5′ end the nucleotide sequence CAT (which creates an NdeI restriction site in combination with the downstream start codon) and at the 3′ end the nucleotide sequence 5′-GGATCCGACTTGGAAGTACTGTTTCAGGGTCCCTAACTCGAG-3′ that encodes a short linker consisting of the amino acids GSD, a 3C protease-cleavage site (amino acids LEVLFQGP), a TAA stop codon and a XhoI restriction site. The complete backbone of vector pET-28b(+) was next amplified by PCR, starting with nucleotides 5′-GCTAACAAA-3′ downstream of the multiple-cloning site and proceeding through nucleotides 5′-GGAGATATA-3′ upstream of the multiple-cloning site. Both PCR amplicons were ligated by Gibson assembly (New England Biolabs). The coding sequence of individual constructs was then inserted immediately downstream of the 3C protease-cleavage site. Truncations and deletions were performed by site-directed mutagenesis (Agilent).

#### Plasmids for recombinant-protein expression in *Pichia pastoris*

Plasmids were assembled in the integrating vector pPICZα-LINK^20^ and are listed in Supplementary Table 2 along with details about their construction and relevant GenBank accession numbers. The vector was first modified to include the coding sequence of GST, followed by a tobacco etch virus (TEV) protease-cleavage site exactly as described previously^24^. The coding sequence of individual constructs was then inserted immediately downstream of the TEV protease-cleavage site by Gibson assembly.

#### Plasmids for genomic integration in *S. cerevisiae*

Plasmids are listed in Supplementary Table 3 along with details about their construction and relevant GenBank accession numbers.

To assemble plasmid MSV-262, the following components were amplified by PCR and ligated by Gibson assembly: a segment of plasmid pWCD1401 located between and including the two NotI restriction sites (containing a high-copy ColE1 origin of replication and bacterial kanamycin-resistance cassette)^54^; the yeast PEX7 coding sequence along with 80 bp of the 5′ untranslated region; the yeast *ADH1* terminator (that is, the last 13 bp of the *ADH1* reading frame along with 190 bp of the 3′ untranslated region) flanked at the 5′ end by the nucleotide sequence 5′-GGCGCGC-3′ (to introduce an AscI restriction site) and at the 3′ end by the nucleotide sequence 5′-GATCCGCTAGATCTCGAGC-3′ (to introduce BglII and XhoI restriction sites); a *hygMX6* hygromycin-resistance cassette (Addgene, catalogue number 19342), followed by an MfeI restriction site; and 80 bp of the 3′ untranslated region immediately downstream of the *PEX7* reading frame. Using site-directed mutagenesis, the nucleotide pair GC was next inserted immediately upstream of the *PEX7* start codon to introduce an SphI restriction site, nucleotides 462 and 468 within the *PEX7* coding sequence were mutated to T and C, respectively, to destroy two BamHI restriction sites and the last four nucleotides of the PEX7 coding sequence (TTGA) were converted to CTAGC to introduce an NheI restriction site.

To assemble plasmid MSV-269, the TAG stop codon in plasmid MSV-262 was replaced by the nucleotide sequence 5′-TCTGGTGATTACAAAGATGATGATGACAAGTAA-3′, which encodes a short linker comprising the amino acids SG, followed by a FLAG tag (amino acids DYKDDDDK) and a new stop codon.

To assemble plasmid MSV-322, the nucleotides GC immediately upstream of the *PEX7* start codon were replaced by a tetracycline-responsive riboswitch (5′-GGCCTAAAACATACCAGATCGCCACCCGCGCTTTAATCTGGAGAGGTGAAGAATACGACCACCTAGGCC-3′)^39^ preceded by a short linker (5′-ATCGTACG-3′) and followed by a minimal Kozak sequence (AAA).

Plasmid MSV-282 was assembled like plasmid MSV-262, with the following modifications. Fragments containing the ColE1 origin of replication and kanamycin-resistance cassette, as well as the *ADH1* terminator region, were amplified by PCR as described earlier. These were ligated by Gibson assembly to the yeast PEX13 coding sequence and 69 bp of the 5′ untranslated region, a *natMX6* nourseothricin-resistance cassette (Addgene, catalogue number 19343) and 68 bp of the 3′ untranslated region downstream of the *PEX13* reading frame. The nucleotide sequence 5′-AGCTCCGAGGATCTGTACTTTCAGAGCTATCCATATGATGTTCCAGATTACGCT-3′, which encodes a linker composed of the amino acids SS, followed by a TEV protease-cleavage site (amino acids EDLYFQS) and an haemagglutinin tag (amino acids YPYDVPDYA), was then inserted immediately upstream of the *PEX13* stop codon. Modifications of the PEX13 coding sequence were performed by site-directed mutagenesis.

Plasmid MSV-324 was assembled like plasmid MSV-282, except using the coding sequence of yeast PEX39, 80 bp of the 5′ and 3′ untranslated regions, and the yeast *LYS2* auxotrophic marker (Addgene, catalogue number 64188)^55^. The nucleotide sequence 5′-GGTTCTGGTGATTACAAAGATGATGATGATAAG-3′, which encodes a linker composed of the amino acids GSG, followed by a FLAG tag, was then inserted immediately upstream of the *PEX39* stop codon.

### Yeast strains and growth conditions

Yeast strains were routinely cultivated on YPD medium (1% wt/vol yeast extract, 2% wt/vol peptone and 2% wt/vol dextrose) at 30 °C, unless stated otherwise; for plates, 2% wt/vol agar (Becton Dickinson, catalogue number 214010) was added to the medium.

*P. pastoris* strains were derived from the SMD1168 parental strain^56^ and are listed in Supplementary Table 4. Expression cassettes for PEX7 and PEX7–GFP were inserted at the *AOX1* locus by homologous recombination using the integrating plasmids described earlier. Plasmids were linearized using the restriction enzyme PmeI (New England Biolabs) and delivered into the yeast cells by electroporation. Transformants were selected on YPD agar supplemented with 1 M sorbitol and 500 µg ml^−1^ zeocin (Thermo Fisher, R25001). Correct insertion was validated by PCR.

*S. cerevisiae* strains were derived from the BY4742 reference strain^57^ and are listed in Supplementary Table 5. Deletions and insertions were performed by homologous recombination in a BY4742 derivative (referred herein as strain MSB-462) that expresses the peroxisomal PTS1 enzyme FOX2 fused at the N terminus to red fluorescent protein (RFP–FOX2) as well as the peroxisomal PTS2 enzyme POT1 fused at the C terminus to green fluorescent protein (POT1–GFP)^58^. For gene deletions, the appropriate antibiotic-resistance cassette or auxotrophic marker was amplified by PCR from the plasmids listed in Supplementary Table 3 using primers that introduce 60-bp overhangs corresponding to the 5′ and 3′ untranslated regions immediately upstream and downstream of the target open reading frame, respectively. The resulting amplicon was delivered into the yeast cells by lithium acetate-based transformation^59^. Transformants were selected on YPD agar supplemented with 250 µg ml^−1^ hygromycin (Thermo Fisher, catalogue number 10687010), 500 µg ml^−1^ geneticin (Thermo Fisher, catalogue number 10131035) or 100 µg ml^−1^ nourseothricin (Jena Bioscience, AB-101), as appropriate, or on synthetic defined agar medium lacking the appropriate amino acid or nucleotide (Sunrise Science). For inserting mutant genes or epitope tags, the relevant reading frame was first knocked out and then replaced by the modified coding sequence using the integrating plasmids described earlier. The plasmids were linearized using the restriction enzyme NotI (New England Biolabs) before lithium acetate-based transformation. All deletions and insertions were validated by PCR.

### Protein purification

#### Purification of PEX7 and PEX7–GFP

PEX7 and PEX7–GFP were produced in *P. pastoris* as fusions to an N-terminal GST tag and a TEV protease-cleavage site, and purified by glutathione-affinity and size-exclusion chromatographies as described previously^24^ with the following modifications. Cells were cultured in 2-l baffled flasks for 36 h at 30 °C on an orbital shaker in BM medium (100 mM KH_2_PO_4_·KOH pH 6.0 at RT, 0.34% wt/vol yeast nitrogen base, 1% wt/vol ammonium sulfate and 4 × 10^−5^% wt/vol d-biotin) supplemented with 1% vol/vol glycerol. The cells were next sterilely resuspended in BM medium supplemented with 0.75% vol/vol methanol in clean 2-l baffled flasks and cultured for an additional 24 h at 28 °C. The final cell pellets were weighed and suspended (at a ratio of 1 ml g^−1^ wet cell weight) in lysis buffer (30 mM Tris–HCl pH 7.5 at RT, 500 mM NaCl, 5% wt/vol glycerol, 1 mM EDTA and 2 mM dithiothreitol (DTT)) supplemented with protease-inhibitor tablets (Roche, catalogue number 5056489001) according to the manufacturer’s instructions. The cell suspension was divided among 50-ml beadbeater canisters (BioSpec, catalogue number 110803-50SS), supplemented with 2 mM phenylmethylsulfonyl fluoride, and the cells were lysed by beadbeating at 4 °C with 0.5-mm glass beads (BioSpec, catalogue number 11079105). The lysate was supplemented with 0.05% wt/vol Tween 20 and clarified by centrifugation at 40,000*g* and 4 °C for 40 min. The resulting supernatant was incubated overnight at 4 °C with glutathione agarose (Prometheus). The beads were washed with excess wash buffer (30 mM Tris–HCl pH 7.5 at RT, 300 mM NaCl and 1 mM DTT) and bound protein was eluted at RT with elution buffer (30 mM HEPES·KOH pH 7.8 at RT, 150 mM NaCl, 5% wt/vol glycerol, 1 mM DTT, 0.5 mM EDTA and 20 mM reduced glutathione). To remove the GST tag, the eluate was mixed with homemade His-tagged TEV protease^60^ and dialysed overnight at 4 °C against elution buffer lacking glutathione. The dialysed solution was incubated with glutathione agarose and Ni-NTA agarose (Thermo Fisher) to adsorb the GST tag and TEV protease. The resulting flow-through was gel-filtered into 30 mM HEPES·NaOH (pH 8), 300 mM NaCl, 5% wt/vol glycerol and 1 mM DTT (at 4 °C) either on a Cytiva HiLoad 16/600 Superdex 75 prep-grade (for PEX7) or a Cytiva HiLoad 16/600 Superdex 200 PG (for PEX7–GFP) column. Peak fractions were pooled and concentrated before being snap-frozen and stored as single-use aliquots at −80 °C.

#### Purification of the YG-domain fragment of PEX13

The unstructured N-terminal region (including the YG domain) of *A. thaliana* PEX13 was produced in *E. coli* BL21 Rosetta 2(DE3) cells (Novagen) as a fusion to an N-terminal 14×His tag and a TEV protease-cleavage site, and purified under denaturing conditions in 8 M urea as previously described^17^.

#### Purification of all other recombinant proteins

Proteins were produced in *E. coli* BL21 Rosetta 2(DE3) cells as fusions containing an N-terminal GST tag and a 3C protease-cleavage site. The proteins were purified by glutathione-affinity and size-exclusion chromatographies as described previously^15^ with the exception that after proteolytic removal of the GST tag using 3C protease, the proteins were gel-filtered into 25 mM HEPES·NaOH pH 7.8 at RT, 150 mM NaCl, 5% wt/vol glycerol and 1 mM DTT before being snap-frozen as single-use aliquots.

### Peptide synthesis

Lyophilized peptides (Genscript) were dissolved in 1 M HEPES·NaOH pH 7.5 at RT, 10 mM tris(2-carboxyethyl)phosphine (TCEP) and 1 mM EDTA to a final concentration of 10 mM, snap-frozen as single-use aliquots and stored at −80 °C. The KPWE peptide corresponds to the amino-acid sequence CSSTAVPRPKPWETSASLEE consisting of residues 2–20 of *S. cerevisiae* PEX13 and an additional N-terminal cysteine for coupling to thiol-reactive agarose beads. In the scrambled peptide, amino acids 8 and 10–13 were rearranged into the sequence CSSTAVPEPRWKPTSASLEE. The PTS2 peptide corresponds to the amino-acid sequence SQRLQSIKDHLVESRS consisting of residues 2–15 of the *S. cerevisiae* peroxisomal thiolase POT1, followed by the amino acids R and S to improve solubility.

### Peroxisome induction

Peroxisome biogenesis was induced in *S. cerevisiae* using an established procedure^61^. Briefly, cells were subcultured twice in YPD medium to mid-log phase (optical density at 600 nm (OD_600_) ≈ 2), harvested through centrifugation at 2,000*g* and 30 °C for 10 min, sterilely resuspended to an OD_600_ of 0.5 in YPGO medium (1% wt/vol yeast extract, 2% wt/vol peptone, 2% vol/vol glycerol, 0.1% wt/vol oleic acid and 0.25% vol/vol Tween 40) and cultured for an additional 16 h at 30 °C. For experiments involving PEX7 riboswitch constructs, cultures in YPGO were supplemented with 250 µM tetracycline (Millipore Sigma, T7660) as described previously^62^.

### Peroxisomal protein import assay in *S. cerevisiae*

*S. cerevisiae* cells expressing RFP–FOX2 and POT1–GFP (MSB-462) were induced to form peroxisomes as described earlier. A 1-ml aliquot of each culture was sedimented by centrifugation for 30 s at 13,000*g* and RT, and the cells were resuspended in 7 µl of RT complete synthetic defined medium (Sunrise Science, catalogue number 1001) supplemented with 10 mM potassium phosphate pH 6 at RT, 4 mM magnesium sulfate, 25 mM ammonium sulfate and 2% wt/vol d-glucose. An aliquot of the resulting suspension was spotted on a clean glass slide (Thermo Fisher, 22-310397), covered by a 22 × 22 mm no. 1.5 square glass coverslip (VWR, 48366-227) and imaged immediately.

The cells were imaged on a spinning-disk confocal platform consisting of a Nikon Eclipse Ti inverted microscope, a Yokagawa CSU-X1 spinning-disk confocal scanner, a Hamamatsu ORCA-Fusion BT sCMOS camera and a Prior Proscan II motorized stage. Samples were illuminated sequentially by solid-state 65 mW 561-nm and 80 mW 488-nm lasers through a Di01-T405/488/568/647 dichroic (Semrock). Fluorescence was collected using a red-emission ET605/52 m (605 ± 26 nm) or a green-emission ET525/50 m (525 ± 25 nm) single-band bandpass filter (Chroma), respectively, through either a Nikon ×100 1.45 numerical aperture (NA) Plan Apo Lambda or Nikon ×40 0.95 NA Plan Apo Lambda air objective. Images were acquired using the Nikon NIS-Elements software with a 16-bit digitizer without binning. Multiple non-overlapping positions arrayed around the centre of the coverslip were imaged for each sample, with focus being maintained at a preset distance from the coverslip using the Nikon PFS. Imaging parameters were configured to maximize the signal-to-noise (SNR) ratio while avoiding saturation. All samples intended to be compared were imaged using identical acquisition settings.

### Preparation of *Xenopus* egg extract

Interphase-locked egg extract lacking filamentous actin was prepared exactly as described in a previously published protocol^37^ using adult female *X. laevis* wild-type pigmented African clawed frogs (aged 20–30 yr; Xenopus1). The frogs were handled strictly within the confines of the Amphibian/Aquatics facility of the Cell Biology Department at Harvard Medical School according to protocols fully approved by the Harvard Institutional Animal Care and Use Committee (protocol number IS00000098-9).

### Peroxisomal protein import assay in *Xenopus* egg extract

Import reactions were performed exactly as described in a previously published protocol^37^. Briefly, extract was mixed with 500 nM of the indicated cargo and 100 nM of the indicated receptor variants with or without 100 nM PEX7, and incubated at 24 °C. Where stated, endogenous PEX5 was first depleted from the extract using agarose beads covalently conjugated to the high-affinity PEX5-binding domain from PEX14. Recombinant proteins were diluted in XBHS buffer (40 mM HEPES·KOH pH 7.7 at RT, 100 mM KCl, 1 mM MgCl_2_, 0.1 mM CaCl_2_ and 250 mM sucrose) before being added to the reactions. For imaging, an aliquot of each reaction was sandwiched between two 22 × 22 mm no. 1.5 square glass coverslips (VWR, 48366-227) that had been passivated with 20 kDa polyethylene glycol (JenKem, M-SLN-20K). The sandwich was mounted on a 25 × 75 mm aluminium slide and sealed with a mixture of Vaseline, lanoline and paraffin. The slides were imaged on a spinning-disk confocal platform consisting of an inverted Nikon Eclipse Ti2 microscope and a Yokagawa CSU-W1 spinning-disk confocal scanner. The samples were illuminated by an 80-mW solid-state 488-nm laser through a Di01-T405/488/568/647 dichroic (Semrock), and fluorescence was collected through a Nikon ×100 1.45 NA Plan Apo Lambda oil-immersion objective and a green-emission ET525/50 m (525 ± 25 nm) single-band bandpass filter (Chroma). Images were acquired using the Nikon NIS-Elements software with a Hamamatsu ORCA-Fusion BT CMOS camera with 2 × 2 binning and a 16-bit digitizer. For each sample, multiple non-overlapping positions arrayed around the centre of the coverslip were imaged using a Prior Proscan III motorized stage operating at 20% of the maximum speed (to avoid shaking the extract). Focus was maintained at a preset distance from the coverslip using the Nikon Perfect Focus System. Imaging parameters were configured to maximize the SNR ratio while avoiding saturation. All samples intended to be compared were imaged under identical acquisition settings.

### Electrophoresis and immunoblotting

Samples in Laemmli buffer (50 mM Tris–HCl pH 6.8 at RT, 10% vol/vol glycerol, 2% wt/vol SDS, 0.01% bromophenol blue and 700 mM β-mercaptoethanol) were heated for 5 min at 95 °C and then electrophoretically resolved under denaturing conditions on 4–20% TGX precast polyacrylamide gels (Bio-Rad, catalogue number 5671095). For immunoblotting, samples were transferred from the gels to Immun-Blot PVDF membranes (Bio-Rad, catalogue number 13709A06) for 12 h at 25 V and RT in 25 mM Tris, 192 mM glycine and 10% vol/vol methanol. The membranes were blotted in 20 mM Tris–HCl pH 7.5 at RT, 150 mM NaCl, 0.01% vol/vol Tween 20 and 3% wt/vol fat-free milk solids (Apex, catalogue number 20241) using antibodies to the FLAG epitope (Sigma, F7425) and compatible horseradish peroxidase-conjugated secondary antibodies (Cytiva, NA934). The blots were developed by chemiluminescence (Perkin Elmer, NEL103001EA) and scanned on an Amersham ImageQuant 800 imaging system. For staining with Coomassie blue, gels were fixed in 50% vol/vol methanol and 10% vol/vol acetic acid for 30 min at RT, incubated for 30 min in 50% vol/vol methanol, 10% vol/vol acetic acid and 0.001% wt/vol Coomassie brilliant blue R-250 (Bio-Rad), and destained overnight in 10% vol/vol acetic acid before washing into water. The stained gels were digitized on an Epson Perfection V700 scanner using the Epson Scan 2 software.

### Cell fractionation and FLAG immunoprecipitation

Cells cultured in YPGO medium (200 ml) were collected by centrifugation at 2,000*g* and 4 °C for 5 min, resuspended in an equivalent volume of water and re-pelleted. The final cell pellet was suspended in 3 ml chilled lysis buffer (20 mM HEPES·KOH pH 6.8 at RT, 150 mM potassium acetate, 5 mM magnesium acetate, 250 mM sorbitol and 1 mM EDTA) supplemented with protease-inhibitor tablets. The cell suspension was divided among three 2-ml screw-cap tubes on ice, supplemented with 1 mM phenylmethylsulfonyl fluoride and lysed by beadbeating with 0.5 mm glass beads on a mini-beadbeater (BioSpec). The cell debris was removed by centrifugation at 2,000*g* and 4 °C for 10 min, and the resulting lysate (2 ml) was further fractionated into a soluble supernatant and a membrane pellet by centrifugation at 20,000*g* and 4 °C for 30 min. A 1-ml aliquot of the supernatant fraction was supplemented with 1% wt/vol highly pure Triton X-100 (Thermo Fisher, catalogue number 85111) and incubated with anti-FLAG agarose beads (Millipore Sigma, A2220) for 1 h at 4 °C. The pellet fraction was gently washed with lysis buffer, resuspended in 2 ml lysis buffer supplemented with 1% Triton X-100, solubilized for 15 min on ice and clarified by centrifugation at 20,000*g* and 4 °C for 10 min. A 1-ml aliquot of the resulting solubilisate was incubated with anti-FLAG beads as described earlier. The beads were washed several times with lysis buffer supplemented with 0.01% Triton X-100 and finally boiled in an equivalent volume of 2×Laemmli buffer.

### YG hydrogel permeation assay

A solution of the purified wild-type YG-domain polypeptide (or the variant lacking the KPWE motif) was concentrated to 2 mM in 50 mM HEPES·NaOH pH 7.8 at RT, 2 M urea and 2 mM TCEP. Aliquots of 2 µl were then spotted on the bottom of multiple wells of a 96-well glass-bottomed plate (Cellvis, P96-1.5H-N), which was then sealed with a gas-impermeable AlumaSeal II film (Hampton Research, HR8-069) to prevent desiccation and incubated at RT for 24 h. Before each assay, the resulting gels were equilibrated overnight at RT in a 100-fold excess of Assay Buffer (25 mM HEPES·NaOH pH 7.8 at RT, 150 mM NaCl and 1 mM TCEP). A 0.5 µM solution of fluorescently labelled protein in Assay Buffer was then added to the samples, with or without additional components at the concentrations indicated in the text, and incubated at RT. Gels were imaged at the times indicated in the text using a Nikon A1R point-scanning confocal system consisting of an inverted Nikon Eclipse Ti microscope and a Nikon ×20 0.75 NA Plan Apo air objective. The objective was centred on the gel edge and focus was maintained 5 µm above the glass surface using the Nikon Perfect Focus System. Fluorescence was excited through a 405/488/561/647 quad-band dichroic (Chroma), using a 488-nm solid-state laser (Coherent) and a galvano scanner. A 50-nm emission band was collected on a gallium arsenide phosphide detector, using an ET525/50m (525 ± 25 nm) single-band bandpass filter (Chroma) and a pinhole dilated to 0.9 Airy units. Images were acquired using the Nikon NIS-Elements software with a pixel size of 1 µm and a 12-bit digitizer. Imaging parameters were configured to maximize the SNR while avoiding saturation. All samples intended to be compared were imaged using identical acquisition settings.

### YG hydrogel release assay

YG hydrogels were prepared in a glass-bottomed plate as described earlier and equilibrated for 3 h at RT in Assay Buffer containing 0.5 µM PEX7–GFP or PEX5–GFP with or without PTS2 peptide and receptor at the concentrations specified in the text. Following the initial incubation, the hydrogels were imaged on a Nikon A1R point-scanning confocal system, as described earlier, with a Nikon ×10 0.45 NA Plan Apo air objective, a pinhole dilated to 1.4 Airy units and a pixel size of 2 µm. A solution of purified PEX39 was then spiked into each reaction and the hydrogels imaged every 2 min for an additional 1 h at RT.

### Bead-binding assays

Cysteine-containing peptides were covalently conjugated to thiol-reactive SulfoLink coupling resin (Thermo Fisher, catalogue number 20401) according to the manufacturer’s instructions. The prepared beads were then mixed with Assay Buffer containing the fluorescently labelled protein as well as additional components indicated in the text, and the resulting bead suspensions were transferred to a 384-well glass-bottomed plate (Cellvis, P384-1.5H-N). GST-fusion proteins were instead mixed with glutathione agarose 4B (Prometheus, 20-543) in Assay Buffer containing the fluorescently labelled protein and additional components indicated in the text; the resulting bead suspensions were then transferred to a glass-bottomed plate as above. The plates were incubated at RT for the durations specified in the text and directly imaged on the spinning-disk confocal platform described earlier for peroxisomal import reactions in *Xenopus* egg extract. Note that imaging the beads directly in the binding reaction preserves the equilibrium and thereby allows even weak interactions to be detected. Fluorescence was collected through a Nikon ×10 0.45 NA Plan Apo air objective, and images were acquired using the Nikon NIS-Elements software and a 16-bit digitizer without binning. Imaging parameters were configured to maximize the SNR while avoiding saturation. Samples intended to be compared were imaged using identical acquisition settings.

### Quantitative binding assays

For the assay shown in Fig. 5d, 15 µl glutathione agarose 4B (Prometheus, 20-543) was mixed with 1 µM purified recombinant yeast PEX7 and varying concentrations of the GST-tagged variants of yeast PEX39, as indicated in the text, in a final volume of 50 µl. Reactions were prepared in Assay Buffer in low protein-binding tubes (Eppendorf, catalogue number 022431081) to minimize sample adhesion to the tube walls. The reactions were incubated at 25 °C for 1 h with agitation. The beads were then quickly sedimented for 30 s at 16,000*g* and RT, and 20 µl of each supernatant fraction was boiled in reducing Laemmli buffer. The samples were resolved by SDS–PAGE and stained with Coomassie blue as described earlier.

For the assay shown in Fig. 8b, the beads were first incubated with yeast PEX7 and the GST-tagged ∆APH variant of yeast PEX39 (each at 1 µM) for 30 min at 25 °C with agitation. Varying concentrations of the peptides, as indicated in the text, were then added and the reactions were incubated for a further 1 h under the same conditions. The beads were finally sedimented and the resulting supernatant fractions resolved by SDS–PAGE as above.

### Statistics and reproducibility

All experiments were repeated independently at least three times. No data were excluded from the analyses. Mathematical fitting was performed in GraphPad Prism using least-squares regression.

### Bioinformatic analysis

Proteins containing KPWE motifs (or conservative variants) were identified in different species using the MOTIF Search tool at GenomeNet (https://www.genome.jp). Amino-acid sequences were aligned using Clustal Omega^63^. Secondary structure predictions were performed with AlphaFold 3 (ref. ^64^). Amino-acid conservation was calculated using ConSurf^65^. Helical-wheel diagrams were prepared using HeliQuest^66^.

### Image analysis

All analyses were performed on the original unmodified images using routine functions in ImageJ^67^. Fluorescence images were first background-subtracted before further analysis.

For the YG hydrogel permeation assay, images were first oriented such that each gel edge faced the same direction. Fluorescence profiles across the buffer–gel interface were then generated by centering a 60 × 300 µm rectangular area on the gel edge and then measuring the mean fluorescence at each position within this selection. To express the resulting values in terms of fold enrichment, the fluorescence at each position along the selection was normalized to the average fluorescence in the buffer and plotted in terms of the distance from the gel edge.

For the PEX7-release assay from YG hydrogels, the total fluorescence within a 30-µm-wide band in the gel extending for 150 µm from the gel edge into the gel was measured at each time point. This distance was sufficient to fully encompass the permeation front of each fluorescent species. The resulting values were then normalized to the total fluorescence inside the gel at the start of the experiment and plotted as a function of time.

To calculate permeation rates through the YG hydrogel, a 60 × 300 µm rectangular area was first centred on the gel edge. The resulting region was then thresholded to demarcate the permeation zone (that is, the distance traversed by the fluorescent species) from the buffer and non-permeated part of the gel. The width of the area above the threshold was then measured and plotted as a function of time. A linear fit to the data yielded the permeation rate.

To calculate import rates in *Xenopus* egg extract, images were first thresholded to identify peroxisomes that were within the focal plane. A circular area with a diameter of 3 pixels was then centred on each local maximum and the mean fluorescence within this area was measured; the average fluorescence in the cytosol was then subtracted from the measured values. The resulting difference (that is, the peroxisome-associated fluorescence) was subsequently converted into a GFP concentration using a calibration curve of GFP fluorescence in the egg extract measured at different concentrations. The peroxisome-associated GFP concentrations were finally plotted as a function of time; linear fits to the data yielded the import rate.

To calculate the fraction bound in the quantitative binding assay, digitized images of Coomassie-stained gels were first converted into 16-bit grayscale and their pixel intensities inverted. The mean pixel intensity was then measured within a rectangular selection encompassing each PEX7 protein band and the mean intensity of the background was subtracted from the measured values. The background-corrected intensities (corresponding to unbound PEX7 remaining in solution) were in turn subtracted from the intensity of the PEX7 band in a sample without PEX39 (that is, total PEX7). The resulting difference (corresponding to PEX7 that had bound to the beads) was finally divided by the total PEX7 intensity to yield the fraction bound. The data were fitted either to a standard binding isotherm or a competitive-binding isotherm, as appropriate, to estimate the observed binding constants.

### Image processing for publication and figure assembly

All images intended to be compared were processed identically. Fluorescence micrographs were normalized to the background before being linearly contrast-stretched in ImageJ to the same bit range. Digitized images of immunoblots and Coomassie-stained gels were linearly contrast-stretched in ImageJ to reveal relevant bands but avoid clipping of the background, whereas photographs of YG hydrogels were processed using Adobe Photoshop. Structural models of proteins were rendered using the PyMOL Molecular Graphics System (Schrödinger). Figures were assembled for publication in Adobe Illustrator.

### Reporting summary

Further information on research design is available in the Nature Portfolio Reporting Summary linked to this article.

## Online content

Any methods, additional references, Nature Portfolio reporting summaries, source data, extended data, supplementary information, acknowledgements, peer review information; details of author contributions and competing interests; and statements of data and code availability are available at 10.1038/s41556-025-01662-5.

## Supplementary information

Reporting Summary

Supplementary Tables 1–5 Plasmids and yeast strains used in this study.

## Source data

Source Data Figs. 2, 5, 6 and 8 and Extended Data Figs. 2 and 9 Numerical source data.

Source Data Fig. 4 Unprocessed immunoblots.

Source Data Fig. 8 Unprocessed gels.

Source Data Extended Data Fig. 1 Unprocessed gels.

Source data Extended Data Fig. 3 Unprocessed gel.

Source Data Extended Data Fig. 7 Unprocessed immunoblots.

Source Data Extended Data Fig. 9 Unprocessed gels and immunoblots.

## Data Availability

Original, unmodified fluorescence images are available from Mendeley Data^68^. Reagents generated by this study are available from the corresponding authors on request. Source data are provided with this paper.
