# Supplementary material for: Import mechanism of peroxisomal proteins with an N-terminal signal sequence
Source: Nat Cell Biol. 2025 May 9;27(6):948–58. doi: 10.1038/s41556-025-01662-5 (PMC12173945; doi:10.1038/s41556-025-01662-5)
Supplement: Supplementary file 1 — Reporting Summary [file 41556_2025_1662_MOESM1_ESM.pdf]

nature portfolio

Corresponding author(s): Tom A. Rapoport
Michael L. Skowyra

Last updated by author(s): 02/22/2025

Reporting Summary

Nature Portfolio wishes to improve the reproducibility of the work that we publish. This form provides structure for consistency and transparency in reporting. For further information on Nature Portfolio policies, see our Editorial Policies and the Editorial Policy Checklist.

Statistics

For all statistical analyses, confirm that the following items are present in the figure legend, table legend, main text, or Methods section.

n/a	Confirmed	
☐	☒	The exact sample size (n) for each experimental group/condition, given as a discrete number and unit of measurement
☐	☒	A statement on whether measurements were taken from distinct samples or whether the same sample was measured repeatedly
☐	☒	The statistical test(s) used AND whether they are one- or two-sided<br>*Only common tests should be described solely by name; describe more complex techniques in the Methods section.*
☒	☐	A description of all covariates tested
☒	☐	A description of any assumptions or corrections, such as tests of normality and adjustment for multiple comparisons
☐	☒	A full description of the statistical parameters including central tendency (e.g. means) or other basic estimates (e.g. regression coefficient) AND variation (e.g. standard deviation) or associated estimates of uncertainty (e.g. confidence intervals)
☒	☐	For null hypothesis testing, the test statistic (e.g. F, t, r) with confidence intervals, effect sizes, degrees of freedom and P value noted<br>*Give P values as exact values whenever suitable.*
☒	☐	For Bayesian analysis, information on the choice of priors and Markov chain Monte Carlo settings
☒	☐	For hierarchical and complex designs, identification of the appropriate level for tests and full reporting of outcomes
☒	☐	Estimates of effect sizes (e.g. Cohen's d, Pearson's r), indicating how they were calculated

Our web collection on statistics for biologists contains articles on many of the points above.

Software and code

Policy information about availability of computer code

Data collection	No custom computer code, algorithm, or software was used.
Data analysis	No custom computer code, algorithm, or software was used.

For manuscripts utilizing custom algorithms or software that are central to the research but not yet described in published literature, software must be made available to editors and reviewers. We strongly encourage code deposition in a community repository (e.g. GitHub). See the Nature Portfolio guidelines for submitting code & software for further information.

Data

Policy information about availability of data

All manuscripts must include a data availability statement. This statement should provide the following information, where applicable:

- Accession codes, unique identifiers, or web links for publicly available datasets
- A description of any restrictions on data availability
- For clinical datasets or third party data, please ensure that the statement adheres to our policy

Source data are provided with this paper. Original, unmodified fluorescence images are available from Mendeley Data. Unique reagents generated by this study are available from the corresponding authors upon request.

Research involving human participants, their data, or biological material

Policy information about studies with human participants or human data. See also policy information about sex, gender (identity/presentation), and sexual orientation and race, ethnicity and racism.

Reporting on sex and gender	Human participants, data, or biological materials were not used in this study.
Reporting on race, ethnicity, or other socially relevant groupings	Human participants, data, or biological materials were not used in this study.
Population characteristics	Human participants, data, or biological materials were not used in this study.
Recruitment	Human participants, data, or biological materials were not used in this study.
Ethics oversight	Human participants, data, or biological materials were not used in this study.

Note that full information on the approval of the study protocol must also be provided in the manuscript.

Field-specific reporting

Please select the one below that is the best fit for your research. If you are not sure, read the appropriate sections before making your selection.

☒ Life sciences ☐ Behavioural & social sciences ☐ Ecological, evolutionary & environmental sciences

For a reference copy of the document with all sections, see nature.com/documents/nr-reporting-summary-flat.pdf

Life sciences study design

All studies must disclose on these points even when the disclosure is negative.

Sample size	All experiments were independently repeated at least 3 times.
Data exclusions	No data were excluded.
Replication	All attempts at replication were successful.
Randomization	Randomization is not relevant to the study because no trial or screen was performed.
Blinding	Blinding is not relevant to the study because all data were acquired under identical acquisition settings and were processed identically prior to analysis by routine automated functions.

Reporting for specific materials, systems and methods

We require information from authors about some types of materials, experimental systems and methods used in many studies. Here, indicate whether each material, system or method listed is relevant to your study. If you are not sure if a list item applies to your research, read the appropriate section before selecting a response.

Materials & experimental systems

n/a	Involved in the study	
☐	☒	Antibodies
☒	☐	Eukaryotic cell lines
☒	☐	Palaeontology and archaeology
☐	☒	Animals and other organisms
☒	☐	Clinical data
☒	☐	Dual use research of concern
☒	☐	Plants

Methods

n/a	Involved in the study	
☒	☐	ChIP-seq
☒	☐	Flow cytometry
☒	☐	MRI-based neuroimaging

Antibodies

Antibodies used	FLAG antibody, Millipore-Sigma, catalog no. F7425
Validation	Comparison to a non FLAG-tagged version of the same protein.

Animals and other research organisms

Policy information about studies involving animals; ARRIVE guidelines recommended for reporting animal research, and Sex and Gender in Research

Laboratory animals	Adult female Xenopus laevis wild-type pigmented African clawed frogs aged approximately 20 years.
Wild animals	The study did not involve wild animals.
Reporting on sex	Only female frogs were used because the study involved the collection of frog eggs for the preparation of frog-egg extract.
Field-collected samples	The study did not involve samples collected from the field.
Ethics oversight	The Harvard Institutional Animal Care and Use Committee (protocol no. IS00000098-9)

Note that full information on the approval of the study protocol must also be provided in the manuscript.

Plants

Seed stocks	Plants were not used in this study.
Novel plant genotypes	Plants were not used in this study.
Authentication	Plants were not used in this study.
