# Supplementary figures and images for: Import mechanism of peroxisomal proteins with an N-terminal signal sequence
Source: Nat Cell Biol. 2025 May 9;27(6):948–58. doi: 10.1038/s41556-025-01662-5 (PMC12173945; doi:10.1038/s41556-025-01662-5)

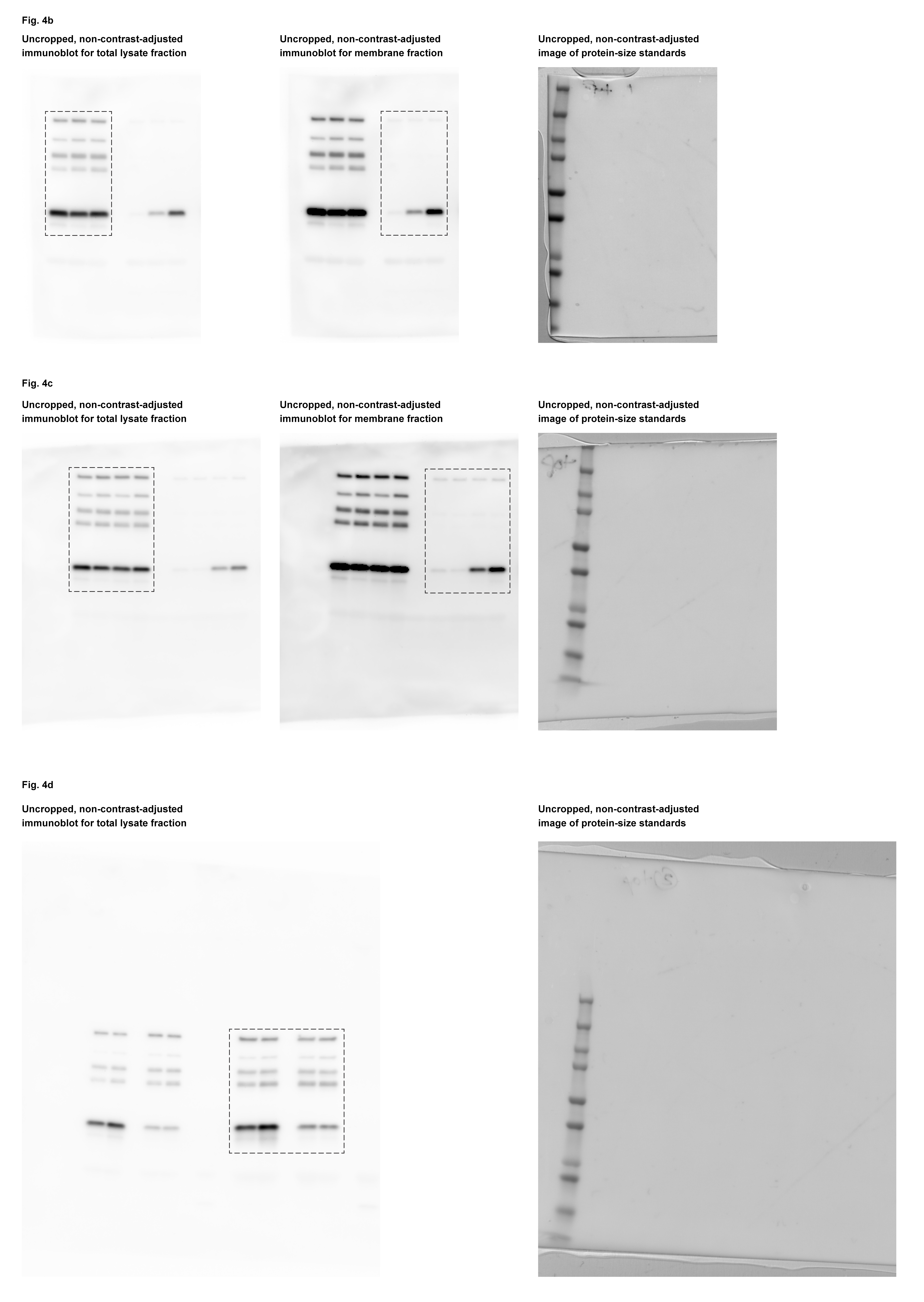

Supplement: Supplementary file 4 — Unprocessed immunoblots. [file 41556_2025_1662_MOESM4_ESM.jpg]

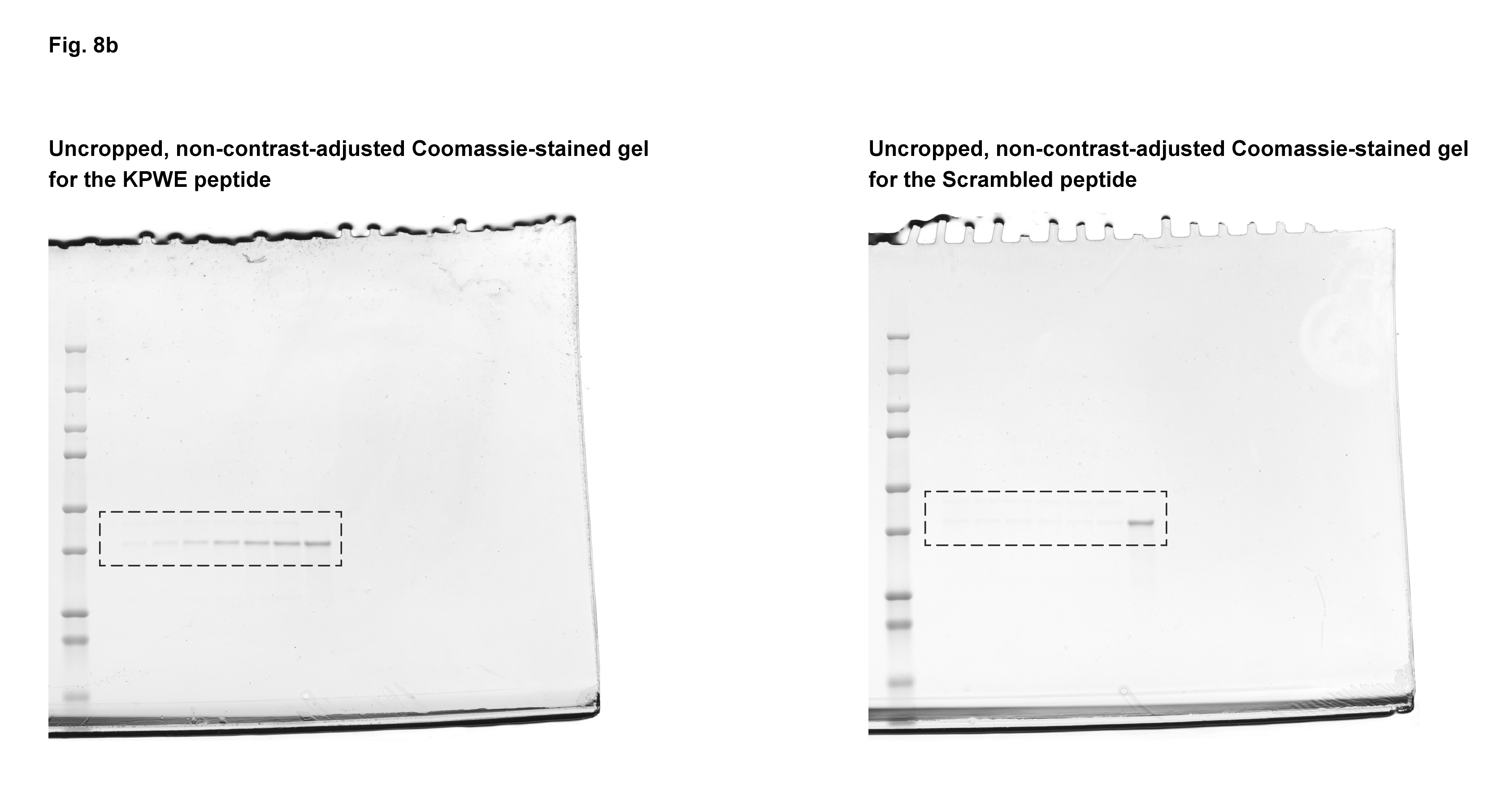

Supplement: Supplementary file 5 — Unprocessed gels. [file 41556_2025_1662_MOESM5_ESM.jpg]

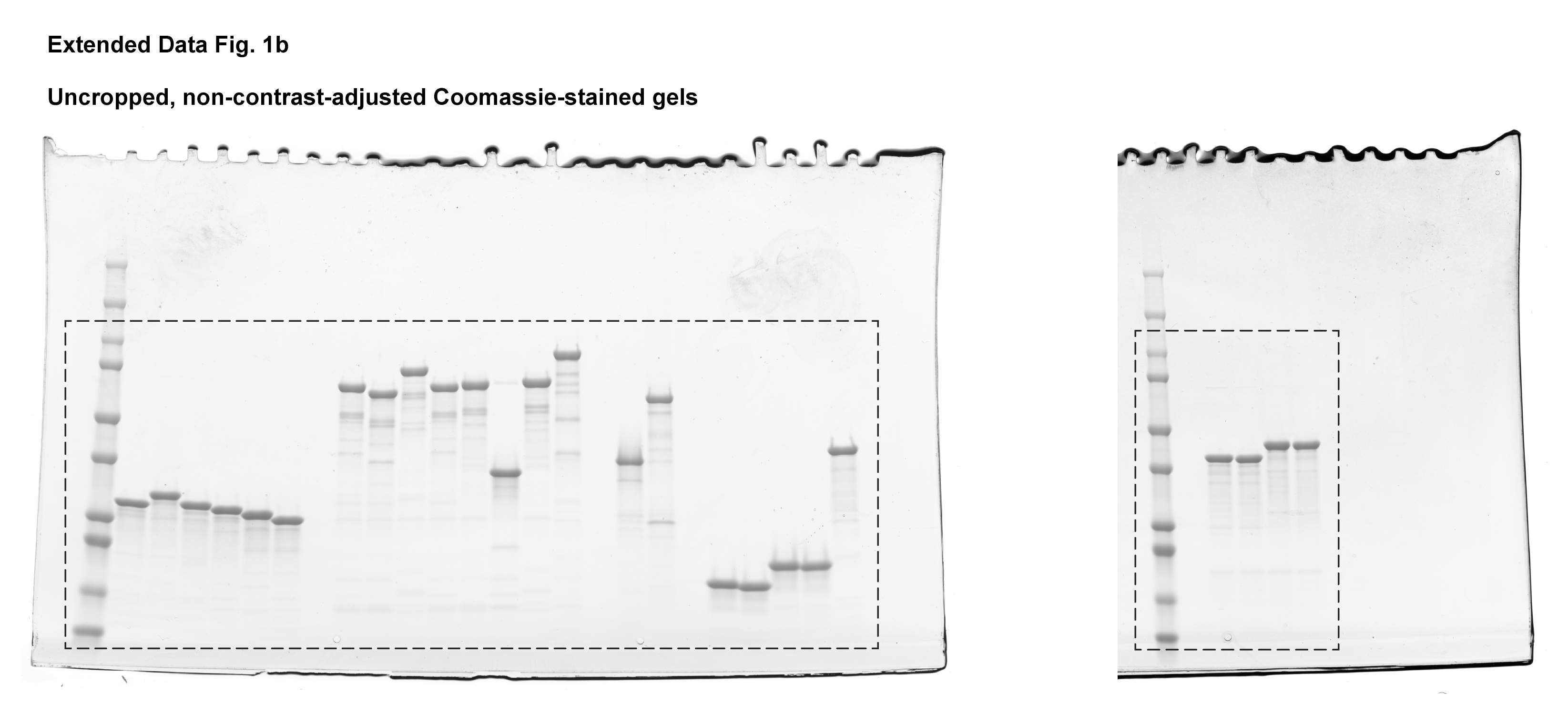

Supplement: Supplementary file 6 — Unprocessed gels. [file 41556_2025_1662_MOESM6_ESM.jpg]

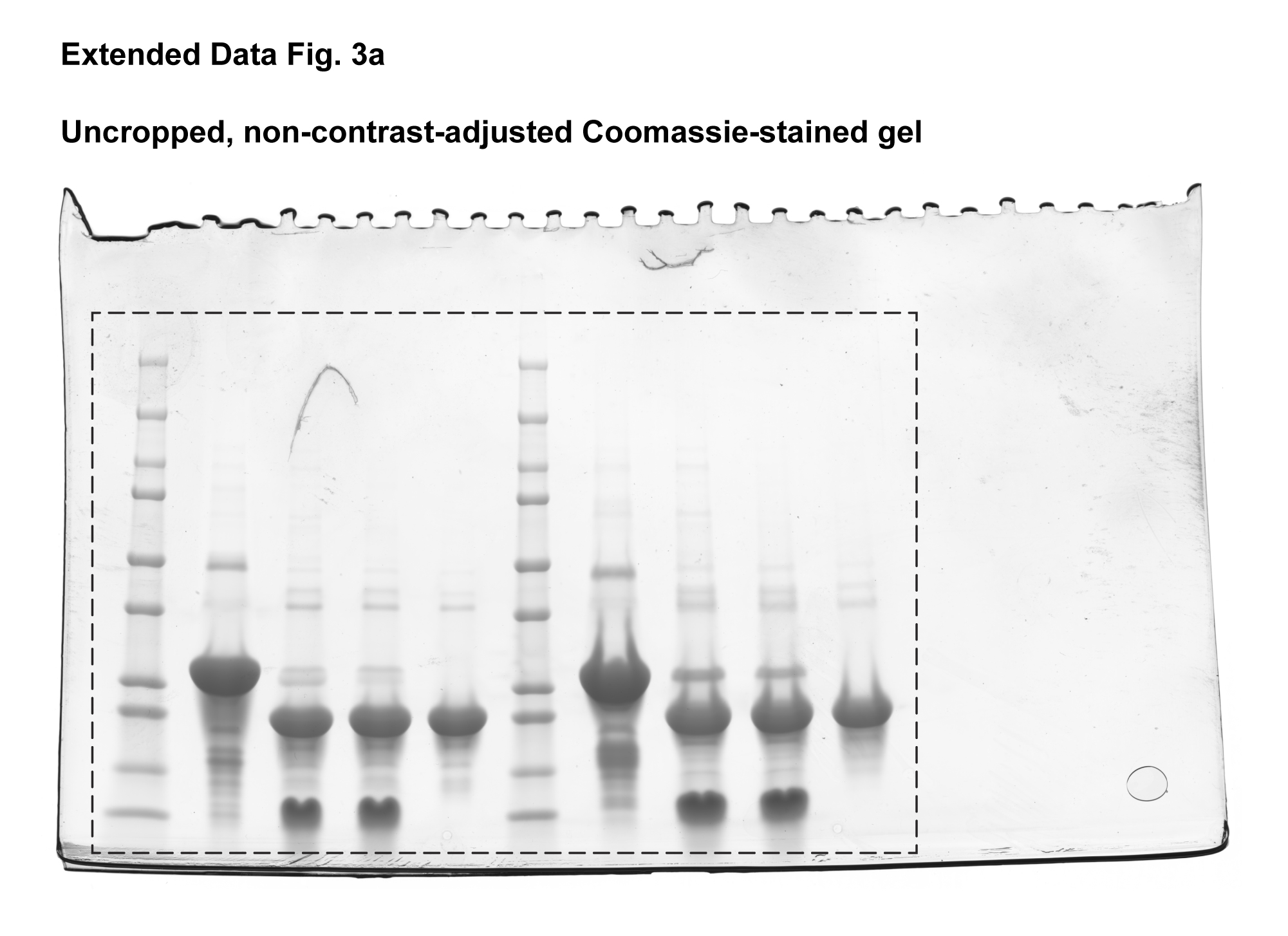

Supplement: Supplementary file 7 — Unprocessed gel. [file 41556_2025_1662_MOESM7_ESM.jpg]

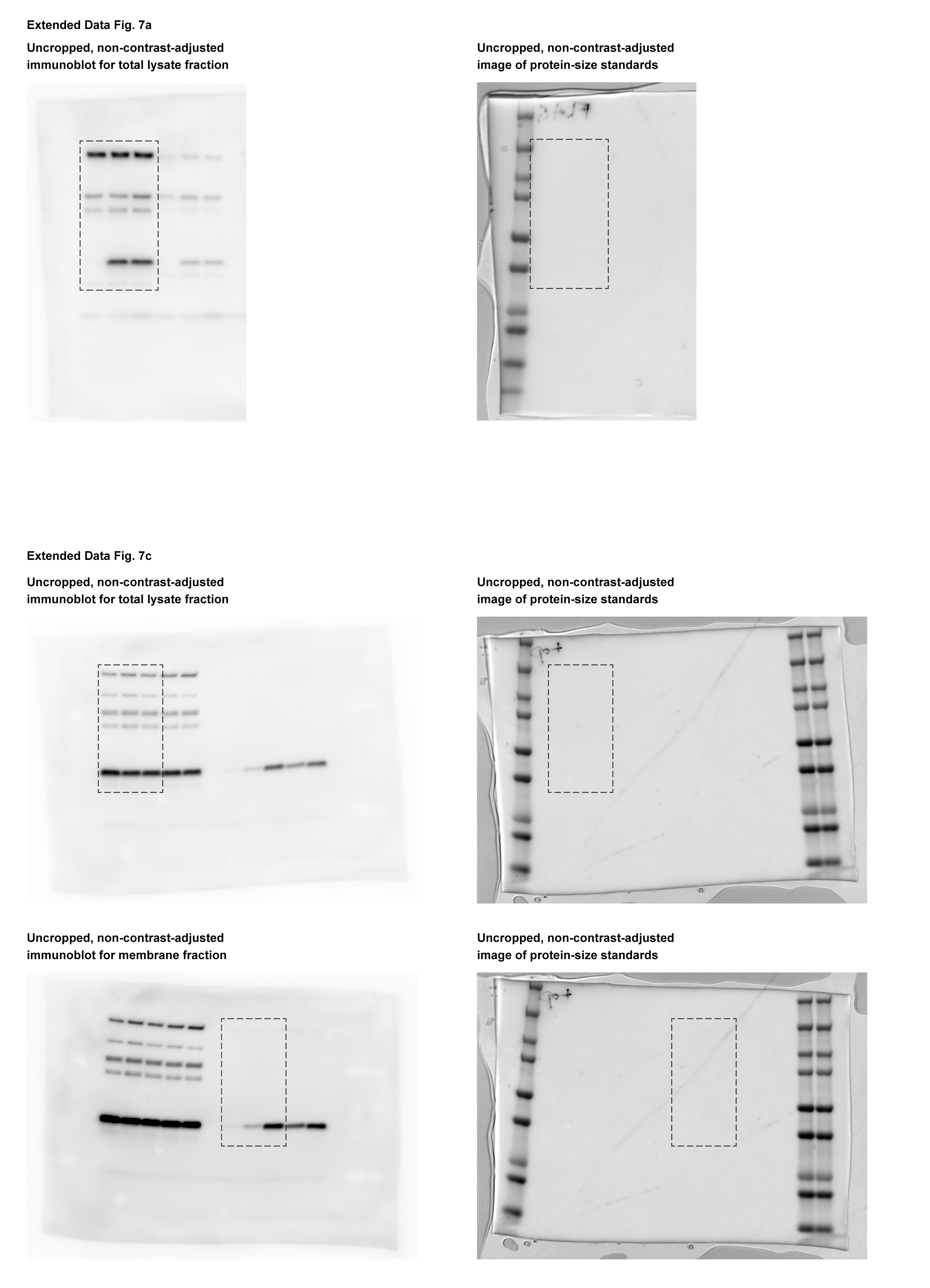

Supplement: Supplementary file 8 — Unprocessed immunoblots. [file 41556_2025_1662_MOESM8_ESM.jpg]

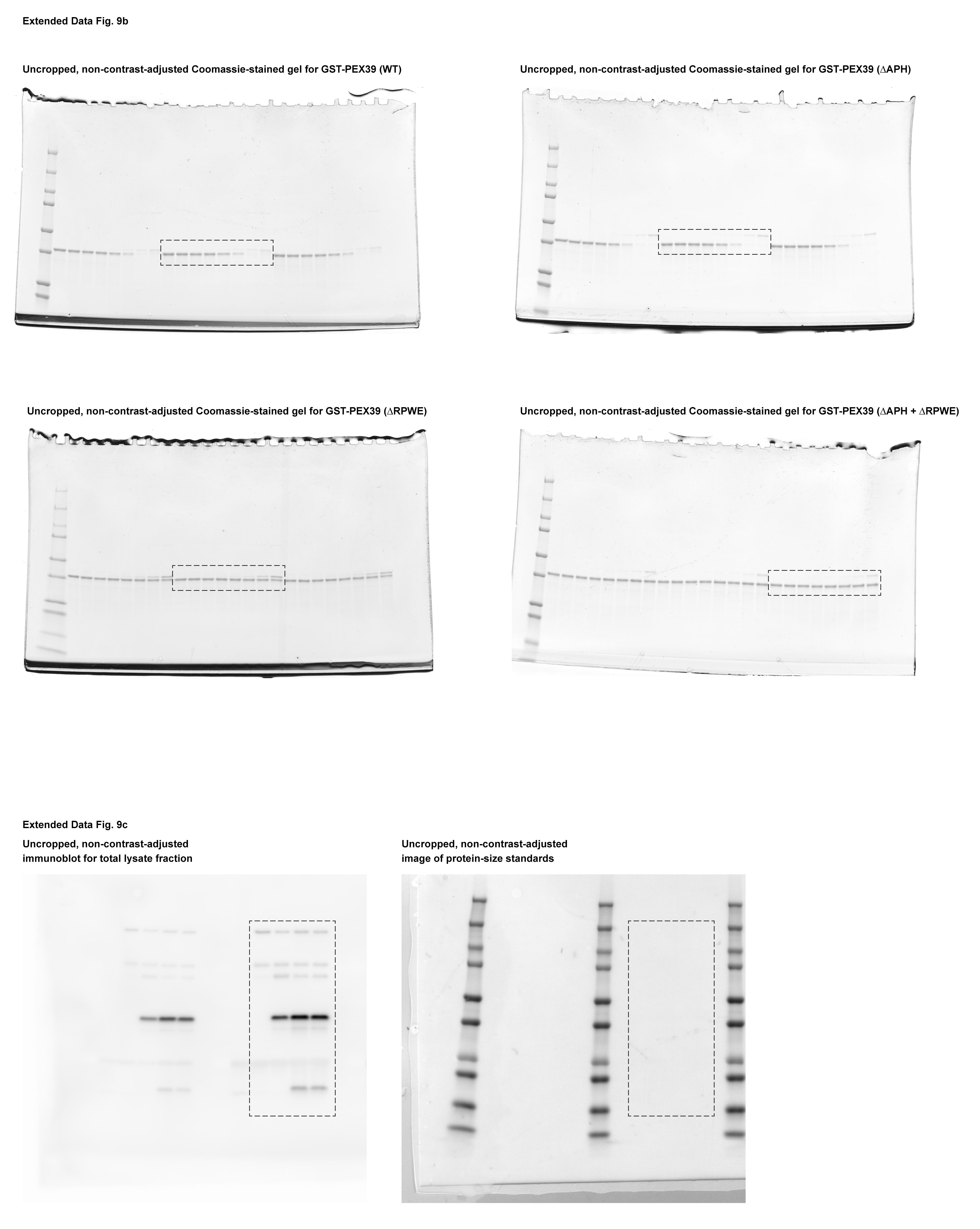

Supplement: Supplementary file 9 — Unprocessed gels and immunoblots. [file 41556_2025_1662_MOESM9_ESM.jpg]
